# Revealing Local Grain Boundary Chemistry and Correlating it with Local Mass Transport in Mixed‐Conducting Perovskite Electrodes

**DOI:** 10.1002/smll.202404702

**Published:** 2024-10-04

**Authors:** Zijie Sha, James O. Douglas, Lluís Yedra, Ieuan D. Seymour, Sònia Estradé, Francesca Peiró, Stephen J. Skinner, John A. Kilner

**Affiliations:** ^1^ Department of Materials Imperial College London London SW7 2AZ UK; ^2^ Laboratory of Electron Nanoscopies (LENS) Department of Electronics and Biomedical Engineering Universitat de Barcelona c/ Marti Franquès 1 Barcelona 08028 Spain; ^3^ Institute of Nanoscience and Nanotechnology (IN2UB) Universitat de Barcelona Diagonal 645 Barcelona 08028 Spain; ^4^ Advanced Centre for Energy and Sustainability (ACES) Department of Chemistry University of Aberdeen Aberdeen Scotland AB24 3UE UK

**Keywords:** ceramics, grain boundaries, mixed ionic electronic conductors, oxygen diffusion, solid oxide fuel cells

## Abstract

Grain boundary (GB) mass transport, and chemistry exert a pronounced influence on both the performance and stability of electrodes for solid oxide electrochemical cells. Lanthanum strontium cobalt ferrite (LSCF6428) is applied as a model mixed ionic and electronic conducting (MIEC) perovskite oxide. The cation‐vacancy distribution at the GBs is studied at both single and multi‐grain scales using high‐resolution characterization techniques and computational approaches. The accumulation of oxygen vacancies ($V_{O}^{\cdot \cdot }$) in the GB region, rather than necessarily at the GB core, results in an enhancement of the oxygen diffusivity by 3 – 4 orders of magnitude along the GBs (*D*_gb_). At 350 °C, the oxygen tracer diffusion coefficient (D*) is measured as 2.5 × 10^−14^ cm^2^ s^−1^. The *D*_gb_ is determined to be 2.8 × 10^−10^ cm^2^ s^−1^ assuming a crystallographic GB width (δ_crystal_) of 1 nm, and 2.5 × 10^−11^ cm^2^ s^−1^ using a chemically measured δ_chem_ of 11.10 nm by atom probe tomography (APT). The origin of the concomitant changes in the cation composition is also investigate. In addition to the host cations, strong Na segregation is detected at all the GBs examined. Despite the low (ppm) level of this impurity, its presence can affect the space charge potential (Φ_0_). This, in turn, will influence the evolution of GB chemistry.

## Graphical abstract

The grain boundary (GB) mass transport and GB chemistry are investigated for La_0.6_Sr_0.4_Co_0.2_Fe_0.8_O_3‐δ_ (LSCF6428), a perovskite oxide electrode. The factors influencing the local cation‐vacancy distribution at the GBs are identified, and this chemical evolution is found to have a pivotal impact on the oxygen transport properties and stability of the electrodes.

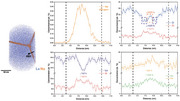

## 1 Introduction

Solid oxide cells (SOCs) are a sustainable electrochemical energy conversion technology based on oxygen ion transport. Nowadays, cells with operating temperature above 650 °C (high temperature) and between 550 and 650 °C (intermediate temperature) are robust and commercially available. However, the elevated operating temperatures have given rise to significant issues, including pronounced performance degradation rates, increased system costs, and slow start‐up and shutdown cycles, thereby impeding the development and widespread implementation of the SOC technology.^[^ ^1^ ^]^ This has initiated research efforts aimed at lowering the operating temperature below 500 °C in order to limit performance degradation, reduce costs associated with insulation and interconnects, and improve the maximum theoretical efficiency.^[^ ^1^ ^]^

There are three major components in a SOC: two electrodes, the cathode and the anode, separated by an oxygen ion conducting electrolyte. Mixed ionic and electronic conducting (MIEC) perovskite oxides (ABO_3_) are extensively employed as electrode materials due to their desirable electrocatalytic activities, ionic and electronic conductivity, affordability, as well as chemical and redox stability.^[^ ^2^ ^]^ The concerns regarding temperature reduction are particularly focused on the cathode and electrolyte, as they have a major contribution to the inefficiencies at low temperatures.^[^ ^3^ ^]^ The key to enabling operation within a low temperature range lies in the acceleration of the oxygen reduction reaction in the cathode, and the enhancement of oxygen transport in both the cathode and electrolyte. Over the past decade, there has been a rapid growth of interest in utilizing grain boundary (GB) transport as a means of improving electrode and electrolyte performance at lower temperature.^[^ ^4^, ^5^, ^6^, ^7^, ^8^, ^9^, ^10^, ^11^, ^12^, ^13^, ^14^ ^]^ Simulation and experimental studies have demonstrated a significant difference in oxygen transport properties along GBs (*D*_gb_), compared to those in adjacent crystals (lattice diffusion, *D*_b_), in both polycrystalline thin film and ceramic samples. For solid electrolytes such as yttria‐stabilized zirconia (YSZ) and gadolinium‐doped ceria (GDC), several orders of magnitude higher GB resistance has been observed compared to the bulk.^[^ ^15^ ^]^ This indicates oxygen ion blocking at the GBs due to excess positive charge, resulting in the depletion of oxygen vacancies ($V_{O}^{\cdot \cdot }$) in the adjacent grains. Various methods have been reported in the literature to improve GB conductivity, such as introducing dopants with lower valences than the host cations^[^ ^13^ ^]^ and illuminating with above‐bandgap light.^[^ ^14^ ^]^ On the other hand, for MIEC conductors, where space charge effects at GB are less critical than in ionic conductors due to the presence of electrons, orders of magnitude enhancement in *D*_gb_ for oxygen have been observed compared to lattice diffusivity.^[^ ^4^, ^5^, ^6^, ^7^, ^8^, ^9^, ^10^, ^11^, ^12^ ^]^ This phenomenon, known as faster GB diffusion, was observed and studied in the range where lattice diffusion rates are below 10^−11^ to 10^−12^ cm^2^s^−1^. Example includes La_0.8_Sr_0.2_MnO_3±δ_ that has slow lattice oxygen transport at all temperatures below 1000 °C,^[^ ^12^ ^]^ and La_0.6_Sr_0.4_Co_0.2_Fe_0.8_O_3‐δ_ (LSCF6428), which exhibits rapid GB oxygen transport at lower temperatures (T <500 °C).^[^ ^6^ ^]^ In addition to the fast‐moving oxygen anions, GBs can also provide preferential paths for the diffusion of slow‐moving cations.^[^ ^16^, ^17^, ^18^ ^]^ It has been demonstrated and suggested that GBs can facilitate the segregation of acceptor dopant (e.g., $Sr_{La}^{\prime }$) toward the surface.^[^ ^16^, ^18^, ^19^ ^]^ Sr surface segregation is one of the major factors contributing to the degradation of electrode materials.^[^ ^20^ ^]^

**Figure 1:**
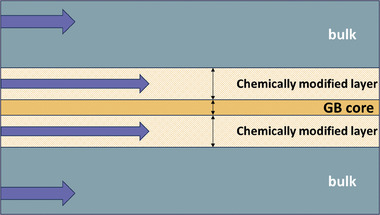
Schematic of accelerated mass transport along chemically modified layers (CMLs) at the GB region, in comparison to the bulk of a perovskite oxide electrode. This occurs due to the accumulation of point defects driving diffusion within the CMLs. The GB is modeled as a regular array of slabs.

Despite promising results, fundamental understanding of GB transport in ionic solids is still lacking. First, the *D*_gb_ values reported in the literature are mainly obtained through the isotopic exchange depth profiling technique (IEDP) coupled with secondary ion mass spectrometry (SIMS).^[^ ^4^, ^5^, ^6^, ^11^, ^21^, ^22^ ^]^ These measurements yield averaged values by effectively averaging the tracer concentration over a selected 300 × 300 mm^2^ area, containing thousands of grains, each with µm‐scale size, at each depth interval. The mass transport properties of individual GBs are not well understood. Some initial studies on individual GBs of MIEC electrodes were conducted on thin films,^[^ ^18^, ^23^ ^]^ a system dominated by low‐angle GBs with a misorientation angle of less than 10°–15° and consisting of a dislocation network.^[^ ^24^ ^]^ However, in ceramics, GBs are typically dominated by high‐angle orientations with misorientation angles above 10°–15°.^[^ ^24^ ^]^ Whether it is realistic to apply the findings from thin films to ceramic samples with different thermal histories, and whether the GB misorientation angle would affect GB chemistry, remain open questions. Further, the mechanism of GB fast diffusion is commonly attributed to the accumulation of point defects, which are responsible for diffusion in the GB region, resulting in the development of chemically modified layers (CMLs)^[^ ^7^, ^12^, ^14^, ^25^, ^26^, ^27^, ^28^, ^29^, ^30^, ^31^, ^32^, ^33^, ^34^, ^35^, ^36^, ^37^ ^]^ adjacent to the GB core. This mechanism is illustrated in **Figure** **1**.

The width of the GB core, commonly referred to as the crystallographic (or structural) width, represents the extent of the region with disrupted lattice periodicity and typically spans a few lattice constants.^[^ ^38^ ^]^ The determination of the width of the CML varies depending on the experimental method used. It can be investigated through electrical measurements derived from bulk capacitance and GB capacitance, based on the “brick layer model”^[^ ^39^, ^40^ ^]^ However, this method neglects defect interactions. In systems with a dopant and a charge‐compensating defect, the characteristic length for the accumulation of point defects is derived from the Poisson–Boltzmann equation and is known as the Debye length. Recent studies have shown that such expressions are typically applicable only to dilute solutions,^[^ ^28^, ^41^, ^42^ ^]^ which does not apply to heavily substituted materials. Additionally, techniques such as transmission electron microscopy (TEM) and atom probe tomography (APT) allow for direct measurement of the width of the CML on single or multi GB scales. However, whether these results are representative of the diverse GB types and their varying properties within the typical GB population of a ceramic sample remains a topic of ongoing investigation.^[^ ^38^ ^]^ Although definitions and values for the width of the GB region vary, most diffusion studies^[^ ^4^, ^5^, ^6^, ^21^, ^22^, ^43^ ^]^ estimate *D*_gb_ based on the product of *D*_gb_ and the width, which is typically assumed to be around 1 nm. The question of whether the assumed or defined crystallographic width accurately represents the width of the chemically altered GB region remains a subject of debate.^[^ ^35^, ^44^ ^]^ There is also an implication that *D*_gb_ may not be as significant as previously proposed, given that the GB product could partly be attributed to the large variation in width.^[^ ^44^ ^]^ In addition, it is crucial to differentiate GB transport between transverse and parallel directions. Studies have demonstrated that GBs can impede the transverse transport of oxygen in polycrystalline electrolyte materials,^[^ ^33^, ^45^, ^46^, ^47^, ^48^, ^49^ ^]^ which is potentially attributed to the presence of space charge and impurities.

To address the challenges outlined above, this study investigated the GB transport of La_0.6_Sr_0.4_Co_0.2_Fe_0.8_O_3‐δ_ (LSCF6428). LSCF6428 was selected as a model MIEC perovskite oxide due to its widespread popularity as an electrode material in SOCs, as well as the extensive knowledge of its crystal structure and transport properties. The macroscopic oxygen transport properties were first investigated using the IEDP technique coupled with SIMS, demonstrating a fast diffusion pathway at low temperatures. To further understand the characteristics of this pathway, APT and TEM were used to reveal the GB chemistry at both single‐ and multi‐grain scales. The findings and methodology of this study allow for the quantification of chemical composition evolution at GBs, providing crucial understanding and design guidelines in the electrocatalytic activity and durability of electrodes for efficient conversion of energy and fuels.

## 2 Results and Discussion

### 2.1 Oxygen Transport Properties

**Figure 2:**
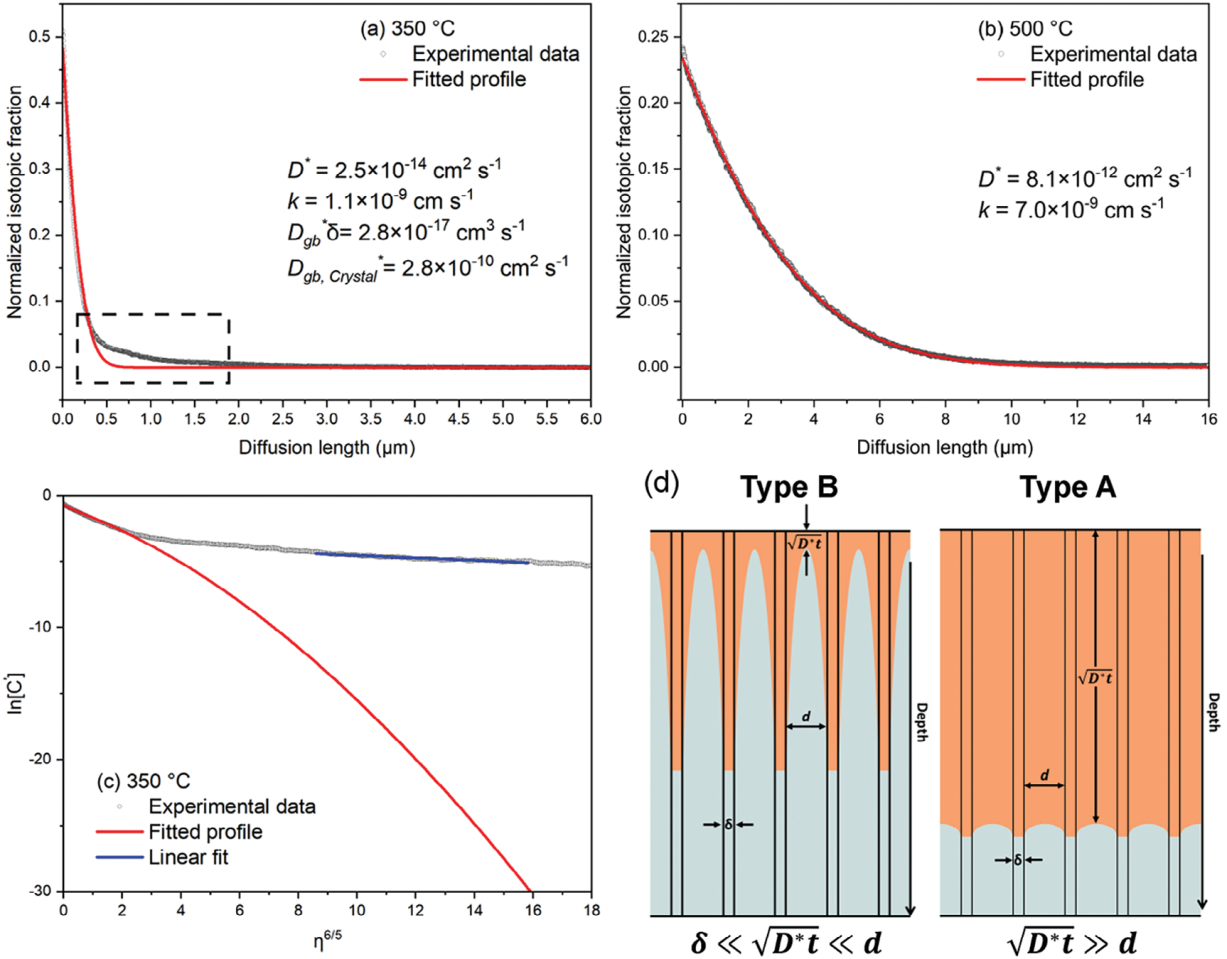
a,b) ^18^O diffusion profiles of LSCF6428 isotopically exchanged at a) 350 and b) 500 °C in the dry oxygen atmosphere (≈1 vppm H_2_O) with pO_2_ of 200 mbar for 3 h. D* represents the oxygen tracer diffusion coefficient, *k* is the surface exchange coefficient, $D_{gb}^{*}\delta$ is the GB diffusion product, and $D_{gb,\;crystal}^{*}$ is the GB diffusivity obtained from the crystallographic GB width, δ_crystal_. c) The diffusion profile at 350 °C plotted as ln[*C’*] against $\eta^{\frac{6}{5}}$, where *C’* represents the normalized isotopic fraction and η represents the normalized depth. d) Schematic illustrations of the Harrison type A and B diffusion regimes^[^ ^50^ ^]^ in a ceramic, depicting grain size (*d*), GB width (δ), and diffusion length ($\sqrt{D^{*}t}$). Diffusion occurs from the top surface toward the bulk and along the GBs.

The ^18^O diffusion profiles of the LSCF6428 samples isotopically exchanged at 350 and 500 °C for 3 h in the dry oxygen atmosphere (pO_2_ = 200 mbar) are illustrated in **Figure** **2**.

In Figure 2a,b, the profiles were collected using SIMS depth profile mode and fitted with Crank's solution to Fick's second law of diffusion for a semi‐infinite medium.^[^ ^51^ ^]^ The obtained oxygen tracer diffusion coefficients (D*) and surface exchange coefficients (*k*) exhibit good agreement with the literature.^[^ ^6^ ^]^ In Figure 2a, a discrepancy ‘tail’ feature, highlighted by the dashed rectangle in the profile collected at 350 °C, demonstrates the existence of fast diffusion pathways for oxygen in the material, such as GBs. According to the Harrison type B^50^ kinetics, as illustrated in Figure 2d and described by Equation 1:

(1) $$20\sqrt{D^{*}t}<d_{g}$$

where *d*_g_ represents the grain size. The bulk diffusion distance should be smaller than the grain size in order to analyze the fast diffusion kinetics. The average grain size of the LSCF6428 was measured to be ≈2.5(1) µm, as demonstrated in the SEM images and the grain size analysis in Figure S1 (Supporting Information). The value of $20\sqrt{D^{*}t}$ obtained at 350 °C remains within the same order of grain size, meeting the criteria for type B diffusion. However, the value obtained at 500 °C significantly exceeds the range corresponding to the Harrison type A^50^ regime, as illustrated in Figure 2d. In this case, the fast diffusion “tail” has disappeared, as shown in Figure 2b. The results also agree with the critical bulk diffusivity value (10^−12^ cm^2^s^−1^) for visibly fast diffusion behavior as provided by Kilner.^[^ ^52^ ^]^

A modified expression for the Crank's solution, as expressed in Equation 2, can be applied to further investigate the potential faster diffusion along the GB at 350 °C:

(2) $$\begin{array}{ccc} & & C^{\prime }\;\left(x,t\right)=\frac{C\left(x,t\right)-C_{bg}}{C_{g}-C_{bg}}\;=\;erfc\left[\frac{x}{2\sqrt{D^{*}t}}\right]\\ & & -\left[\exp\left(\frac{kx}{D^{*}}+\frac{k^{2}t}{D^{*}}\right)\times erfc\left(\frac{x}{2\sqrt{D^{*}t}}+k\sqrt{\frac{t}{D^{*}}}\right)\right]\\ & & +A_{gb}\exp\left(-Z_{gb}x^{\frac{6}{5}}\right) \end{array}$$

In Equation 2, the initial part represents Crank's solution for diffusion in a semi‐infinite medium,^[^ ^51^ ^]^ while the latter part $A_{\mathrm{gb}}\exp\left(-Z_{\mathrm{gb}}x^{\frac{6}{5}}\right)$ is attributed to GB diffusion, where *A*_gb_ and *Z*_gb_ are the parameters describing the GB tailing function. Thus, within the ‘tail’ region of the plot, the diffusion profile is expected to exhibit a linear relationship when plotting ln[*C’*] against *x* ^6/5^, where the slope corresponds to ‐*Z*_gb_. The Le Claire approximation^[^ ^53^ ^]^ is further applied to calculate the potential GB diffusion product ($D_{\mathrm{gb}}^{*}\delta$) based on a relation delineated in Equation 3:

(3) $$D_{\mathrm{gb}}^{*}\delta \;=\;0.66{\left(\frac{4D^{*}}{t}\right)}^{\frac{1}{2}}{Z_{gb}}^{-\frac{5}{3}}\;$$

**Figure 3:**
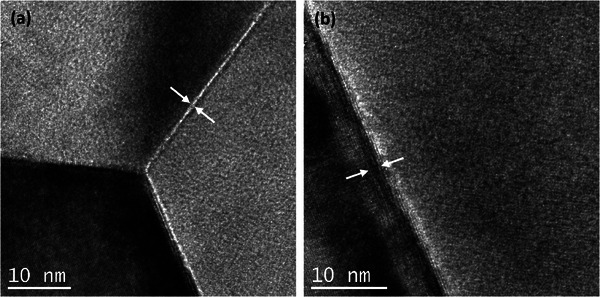
a,b) TEM images of LSCF sample annealed at 350 °C. The white arrows indicate the crystallographic GB width, δ_crystal_. It is noted in Figure 3b that the slanted boundary results in a few unit cells wider measurement.

With respect to the linear relationship, Equation 3 can be further rearranged as:

(4) $$D_{\mathrm{gb}}^{*}\delta \;=\;1.32{\left(D^{*}\right)}^{\frac{3}{2}}t^{\frac{1}{2}}\left[{\left(-\frac{\partial \ln C\left(x,\;t\right)}{\partial \eta^{\frac{6}{5}}}\right)}^{-\frac{5}{3}}\right]$$

where a normalized depth $\eta \;=\;\frac{x}{\sqrt{D^{*}t}}$. Figure 2c shows the diffusion profile plotted as ln[*C’*] against $\eta^{\frac{6}{5}}$, and the gradient was determined in the depth range of 6 ≤ η ≤ 10. The $D_{\mathrm{gb}}^{*}\delta$ was calculated to be 2.8 × 10^−17^ cm^3^s^−1^ using Equation 4. The crystallographic GB width, δ_crystal_, was determined to be ≤1 nm, as evidenced by the TEM images in **Figure** **3**.

Hence, it can be inferred that GB diffusivity obtained from the δ_crystal_, $D_{\mathrm{gb},\;\mathrm{crystal}}^{*}$, is 2.8 × 10^−10^ cm^2^s^−1^, which is four orders of magnitude larger than *D* ^*^ at 2.5 × 10^−14^ cm^2^s^−1^. To understand the characteristics of this notably rapid diffusion pathway and correlate the local chemistry of the GB to local mass transport, APT and TEM were applied at both single GB and multi‐GB scales. The results are discussed in the following section.

### 2.2 Grain Boundary Chemistry

**Figure 4:**
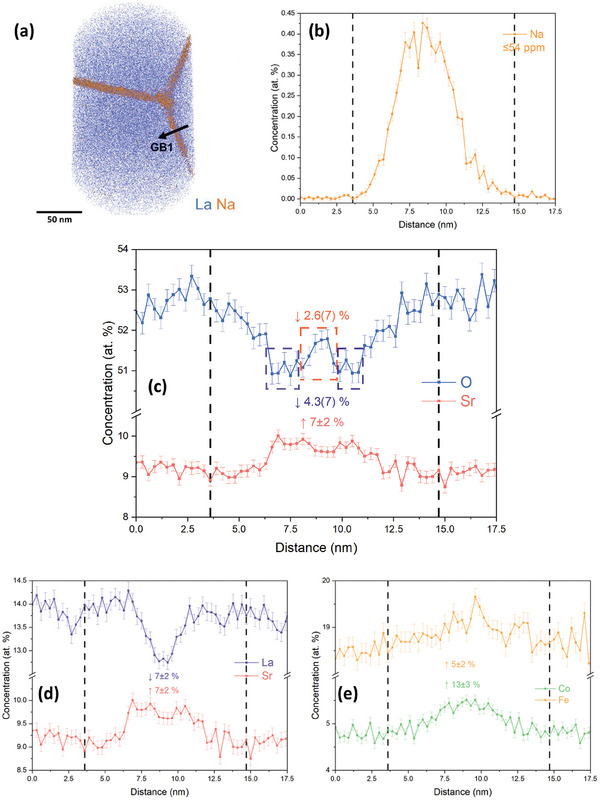
a) APT reconstruction of the LSCF6428 sample annealed at 350 °C. The interaction volume is 50.4 × 133.3 × 18.0 nm^3^. The blue dots represent La matrix atoms, while the orange spheres represent Na, indicating the presence of the GB region. b–e) Elemental distribution profiles of b) Na, c) O versus Sr; d) La versus Sr; and e) Co versus Fe across GB1, as highlighted in Figure 4a.

**Figure** **4** demonstrates an APT reconstruction of a triple GB region obtained from the LSCF6428 sample, which was exchange annealed at 350 °C, along with the corresponding 1D concentration profile across grain boundary 1 (GB1). The feature is located at a depth of 1 µm or deeper.

In Figure 4a, the triple GB region is highlighted by the Na map, revealing a tendency for the impurity to segregate to the GBs. The presence of Na could originate from the commercial LSCF powder or from sintering, annealing, or FIB milling processes. Notably, Xu et al.^[^ ^35^ ^]^ have reported a similar phenomenon where impurities such as Si and Al are likely to accumulate at the GBs of 0.2 cation% Sm‐doped ceria. The total impurity level of Na in the LSCF sample, *f*_Na_, can be estimated from the APT results, as detailed in Section S‐2 of the (Supporting Information). The *f*_Na_ was determined to be ≤54 ppm, as indicated on the Na profile in Figure 4b. Despite impurities at the GBs, this sample is purer than the majority of those summarized in the literature,^[^ ^35^ ^]^ with no detectable impurities in the bulk.

As for the constituent ions, Figure 4c reveals a deficiency in the concentration of O and an enrichment of Sr within the GB region. Notably, the profiles of O and Sr exhibit a complementary shape. The oxygen deficiency exhibited by the GB provides experimental evidence of the segregation of oxygen vacancies, $V_{O}^{\cdot \cdot }$, attributed to their lower formation energy of $V_{O}^{\cdot \cdot }$ at the GBs.^[^ ^36^, ^37^, ^54^, ^55^ ^]^ The general trend for O and Sr in the GB region is consistent with both ab initio atomistic simulations,^[^ ^56^, ^57^, ^58^ ^]^ and experimental studies.^[^ ^18^, ^59^, ^60^ ^]^ However, as indicated by the dashed orange rectangle, an unexpected increase in the concentration of O was observed at the GB core, as defined by the peak of the Na profile in Figure 4b, in contrast to the adjacent regions highlighted by the purple rectangle. This observation contradicts the generally accepted phenomenon for oxygen ion conductors, where $V_{O}^{\cdot \cdot }$ tends to accumulate at the GB core, creating an excess positive charge in the core, followed by chemically modified layers (CMLs) adjacent to the core, where [$V_{O}^{\cdot \cdot }$] is considered to be depleted.^[^ ^7^, ^26^, ^28^, ^37^, ^46^, ^61^ ^]^ Several key points need to be emphasized. First, even though [O] was observed to be higher at the GB core, its concentration remains lower than that of the bulk. The core showed a 2.6(7)% oxygen deficiency and an ≈0.07 deficiency in oxygen non‐stoichiometry, while adjacent regions had a 4.3(7)% oxygen deficiency and an ≈0.13 deficiency in the non‐stoichiometry. The relative values are compared here since the absolute oxygen content is known to be challenging to measure through APT.^[^ ^18^, ^62^, ^63^, ^64^, ^65^ ^]^ It is worth noting that the oxygen concentration displayed in Figure 4c is slightly lower than the stoichiometric value of 60%, while the expected stoichiometry for LSCF annealed in oxidizing environments with high pO_2_ should be closed to O_3_.^[^ ^66^ ^]^ Possible reasons for this deviation include the overlap between O^+^ and O_2_^2+^ at 16 Da, potentially causing some ions to be counted as double or half their ‘real’ value. Oxygen species may evaporate between laser pulses and so may not be detected with the appropriate time of flight to be assigned correctly. Secondly, the noise plot shown in Figure S2a (Supporting Information) indicates the increase of O signal at the core is not attributed to a local increase in noise. The standard errors in Figure 4 were calculated based on the ion counts, and detailed information can be found in Section S‐2 of the (Supporting Information). In addition, the oxygen profile in Figure 4c contains the O signal from all oxygen‐containing species. The profile corresponding to 16 Da, which represents ^16^O as depicted in Figure S2b (Supporting Information), similarly confirms the aforementioned observation. The deficiency of $V_{O}^{\cdot \cdot }$ at the GB core is likely the result of the presence of impurity ions, such as Na, which has been demonstrated to be responsible for the space charge potential,^[^ ^35^ ^]^ which, in turn, affects the composition evolution at the GB. Despite the $V_{O}^{\cdot \cdot }$ accumulation found in GB1, no significant enrichment of the isotopic fraction ($\frac{\left[Mass\;18\right]}{\left[Mass\;18]+[Mass\;16\right]}$) was observed, as demonstrated in Figure S3 (Supporting Information). This could be attributed to the fact that the fast diffusion “tail” feature is only visible at a depth of ≈0.3 to 1.3 µm, as indicated by the diffusion profile in Figure 2a, and GB1 is situated outside this depth range. Higher isotopic fractions at GBs in nanocrystalline thin films of La_0.9_Sr_0.1_CrO_3_^[^ ^23^ ^]^ and La_0.8_Sr_0.2_MnO_3_,^[^ ^18^ ^]^ perovskite oxides with chemical compositions similar to LSCF, have been demonstrated through APT. However, in the case of a ceramic sample with a grain size two orders of magnitude larger (a few µm for the ceramics and tens of nm for the thin films) and a much shallower depth range for the fast diffusion ‘tail,’ identifying a GB with a higher isotopic fraction becomes extremely challenging using APT with tip apex less than 100 nm. Furthermore, the enrichment of Sr in the GB region can also be explained by a space charge scenario, in which charge compensation occurs between the positively charged $V_{O}^{\cdot \cdot }$ and the negatively charged $Sr_{La}^{\prime }$. The Sr concentration was determined to be enriched by 7±2% in the GB region.

Regarding the A‐site cations, as described in Figure 4d, there is a 7 ± 2% of deficiency of La in the GB region, which is complementary to the enrichment level of Sr. Interestingly, an enrichment of B‐site cations, Co and Fe, is also found in the GB region as demonstrated in Figure 4e. The driving force for elemental segregation at the GBs was further studied using density functional theory (DFT) energetics retrieved from the Materials Project Database,^[^ ^67^ ^]^ as detailed in the Section S‐4 (Supporting Information). The simulation was conducted at four temperatures: 0, 350, 500, and 1250 °C, to investigate the effects of sintering and thermal annealing. The simulations consider the decomposition of the material into bulk phases, but they can be used as an approximation for the formation of local GB environments or ‘complexions’.^[^ ^68^ ^]^ It has been demonstrated that the favorable formation of Sr–Co–Fe–O complexions occurs at the GBs. Additionally, at all temperatures, the presence of the impurity Na can also induce the formation of Na–Co–O complexions. The simulation establishes the existence of a thermodynamic driving force for the segregation of B‐site cations at GBs, leading to the formation of complexions with Sr and/or Na. Despite the kinetic likelihood of these complexions forming only during the sintering stage at much higher temperatures,^[^ ^16^ ^]^ a detailed discussion on this matter will follow. Moreover, the observation of B‐site cation enrichment at the GB can be correlated with the formation of the B‐site enriched secondary phase, as previously reported for heat‐treated LSCF under reactive gases at elevated temperatures.^[^ ^69^ ^]^

**Figure 5:**
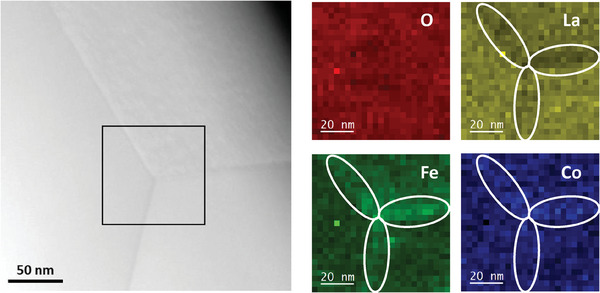
EELS at.% compositional maps collected from LSCF6428 sample annealed at 350 °C, highlighting the triple GB region.

The elemental profiles across another GB, GB2, in the triple GB region are displayed in Figure S4 (Supporting Information). The Na and Sr segregation, O and La deficiency, and Fe enrichment at the GB region, as discussed in the previous paragraph, can be summarized. However, the varying levels of enrichment and deficiency for each element at the GB region emphasize the possible distinct space charge potential and, consequently, the distinct chemistry of each GB. For instance, Figure S4e (Supporting Information) shows no noticeable enrichment of Co, unlike the clear enrichment observed in Figure 4d. Furthermore, similar findings can be extrapolated to a multi‐grain scale, in addition to the individual GBs studied by APT. **Figure** **5** illustrates the EELS quantitative maps for a triple GB region from a different LSCF sample than the one analyzed with APT, while maintaining the same thermal history and undergoing exchange annealing at 350 °C.

In Figure 5, the triple GB region exhibits enrichment in Fe and Co, accompanied by a reduced amount of La, in good agreement with the APT results. Since the deficiency of O probed by APT is less than 5%, it is challenging to demonstrate through EELS mapping as it falls within the quantification uncertainty. EELS is also unable to capture information regarding the distribution of Sr due to the instrument's limitations in detecting the Sr signal at high energy.

**Figure 6:**
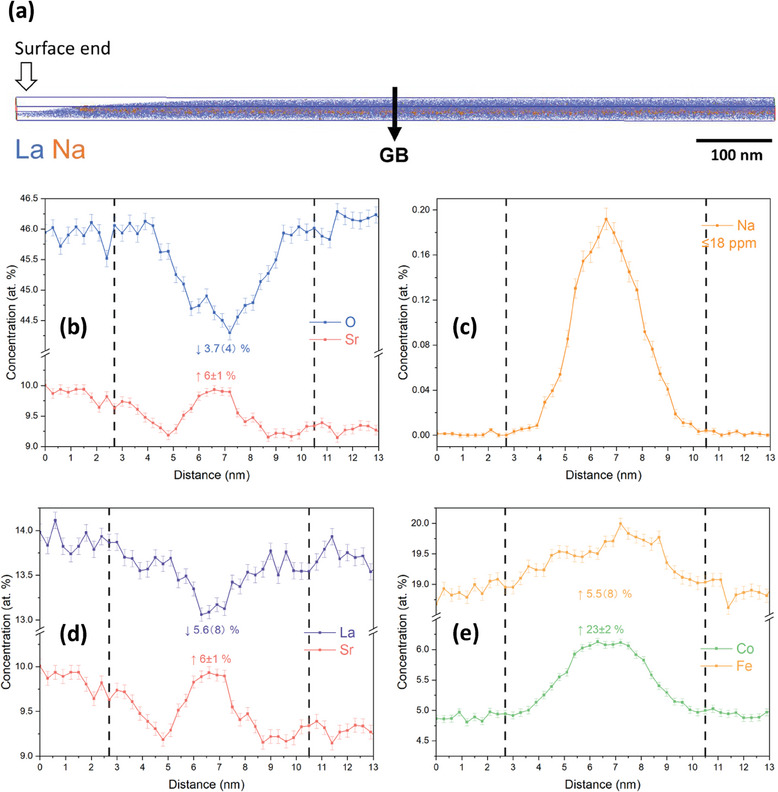
a) APT reconstruction of the LSCF6428 sample annealed at 500 °C. The interaction volume is 14.4 × 45.2 × 629.1 nm^3^. The blue dots represent La matrix atoms, while the orange spheres represent Na, indicating the presence of the GB region. b–e) Elemental distribution profiles of (b) O versus Sr; (c) Na; (d) La versus Sr; and (e) Co versus Fe across the GB.

**Figure** **6** further illustrates the APT reconstruction of a GB located a few µm in depth and extending toward the surface, along with the corresponding elemental profile collected from the LSCF6428 sample annealed at 500 °C.

In Figure 6b, the GB region displays a consistent trend of O deficiency and Sr enrichment. However, the relative deficiency of $V_{O}^{\cdot \cdot }$ at the core, as observed in the GB shown in Figure 4a, was not found. This could be attributed to the lower *f*_Na_ observed on the GB, as indicated by Figure 6c (≤18 ppm), in comparison to the previously found 54 ppm. Despite a higher $V_{O}^{\cdot \cdot }$ level at the GB region, Figure S5 (Supporting Information) indicated no increase in the ^18^O isotopic fraction. The finding is consistent with the diffusion profile depicted in Figure 2b, wherein no GB fast diffusion tail is observed. It is noteworthy that the fast transport of oxygen along specific GBs at a particular depth, as indicated by the “tail” in Figure 2a, predominantly occurs in a Harrison type‐B scenario, characterized by a bulk diffusion distance significantly smaller than the grain size. When the bulk diffusivity of $V_{O}^{\cdot \cdot }$ and oxygen ion increases with temperature, and the oxygen bulk diffusion distance surpasses the grain size, it is indicated that oxygen ions have diffused along and across multiple GBs, described as a Harrison type‐A scenario. In such cases, the observation of faster GB transport diminishes even with the accumulation of $V_{O}^{\cdot \cdot }$ at the GBs, and the diffusion of oxygen ions is considered to occur within a homogeneous medium, resulting in an effective diffusion coefficient.^[^ ^21^ ^]^ This finding aligns well with a recent simulation study by Bonkowski et al.^[^ ^70^ ^]^ on lanthanum strontium ferrites. Further, the increase in Sr also indicates the accumulation of Sr at the GBs. This could suggest that the GB may serve as a fast pathway for transporting Sr toward the surface.^[^ ^16^, ^18^, ^19^ ^]^ The Sr surface segregation, driven by various elastic and electrostatic forces, is a known issue that can directly impact the kinetics of oxygen surface exchange and the chemical stability of the electrodes.^[^ ^20^ ^]^ The Sr concentration on the surface was quantified through XPS, with the results displayed in **Table** **1**.

**Table 1:** The atomic ratio of [Sr]_surface_ [Sr]_lattice_ and [La]_total_: [Sr]_total_ obtained from the as‐polished LSCF6428 sample, as well as from samples annealed in dry oxygen at 350 and 500 °C, as determined through XPS with an approximate 10 nm probe depth.

Sample	[Sr]_surface_:[Sr]_lattice_	[La]_total_:[Sr]_total_
As‐polished LSCF6428	23: 77	63: 37
LSCF6428 annealed at 350 °C	38: 62	62: 38
LSCF6428 annealed at 500 °C	48: 52	59: 41

In Table 1, the atomic ratios of [Sr]_surface_: [Sr]_lattice_ and [La]_total_: [Sr]_total_ represent two vital indicators of the Sr enrichment level on the surface. The [Sr]_surface_: [Sr]_lattice_, obtained from the fitting of the Sr 3*d* spectra demonstrated in Figure S6 (Supporting Information), indicates the relative ratio of Sr in surface species ([Sr]_surface_) and in the perovskite lattice in the near‐surface region ([Sr]_lattice_). The atomic ratio of the La content ([La]_total_) to the Sr content ([Sr]_total_) represents the total Sr concentration on the surface and was deduced from the peak areas of the Sr 3*d*_5/2_ and La 3*d*_5/2_ photoelectron peaks after subtraction of a Shirley‐type background.^[^ ^71^ ^]^ Despite the changes in the chemical environment of Sr, as indicated by the increase in the amount of Sr surface species, [Sr]_surface_, during the annealing process and its elevation with temperature, the total Sr content, [Sr]_total_, remains ≈unchanged within the quantification uncertainty of XPS analysis.^[^ ^72^ ^]^ The diffusivity of Sr, as estimated from the study conducted by Kubicek et al.,^[^ ^16^ ^]^ is presented in Table S1 (Supporting Information). Given the extremely low diffusivity for Sr cations in the bulk and along the GBs at the annealing temperatures of 350 and 500 °C,^[^ ^16^ ^]^ it is plausible that these temperatures have minimal effects on the cation contents on the surface and at the GBs. On the other hand, the rapid Sr diffusion kinetics at 1250 °C suggest that it is highly probable that the cation contents at the GBs were established during the sintering process, in accordance with the previously described *restricted‐equilibrium* scenario.^[^ ^26^, ^37^, ^73^ ^]^ In addition, as the APT analysis was carried out using laser pulsing, there can be variations in field and therefore evaporation behavior across the sample apex with respect to the direction of laser incidence.^[^ ^74^ ^]^ This often exhibits itself through apparent density variations and changes in measured concentrations from the incident side to the non‐incident side.^[^ ^74^, ^75^ ^]^ The plane of the GB in Figure 6a was approximately parallel to the laser incident side. Therefore, the uneven Sr profile on either side of the GB in Figure 6b,d is likely due to this orientation effect, rather than local variations in composition. Sr distributions in other samples of this material without GBs were analyzed and found to follow the same trend.

**Figure 7:**
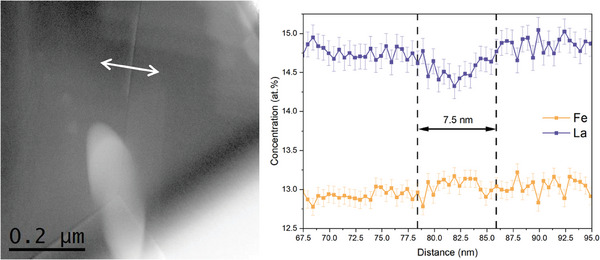
Line‐scan profiles across a GB of the LSCF6428 sample annealed in dry oxygen at 500 °C.

Furthermore, Figure 6d,e demonstrates a consistent relative change in the A‐site and B‐site cations at the GB, respectively. It is acknowledged that inaccuracies may arise in determining the compositional width of an interface, i.e., the chemically defined GB width (δ_chem_), through the APT technique. These inaccuracies result from a complex interplay of factors, such as the variation in field experienced by the boundary with respect to its orientation with analysis direction.^[^ ^76^ ^]^ The combined effects of these factors influence the projection of ions from the apex of the specimen onto the position‐sensitive detector. EELS was applied as a complementary technique to verify the width of the chemically altered GB region, distinguished by La deficiency and Fe enrichment features. The line‐scan profiles, obtained from a different LSCF sample than that analyzed with APT but with the same thermal history, are illustrated in **Figure** **7**.

Figure 7 shows that δ_chem_, measured by EELS, is 7.5 nm, which closely aligns with the 7.80 nm obtained by APT as indicated in Figure 6. It can be concluded that the δ_chem_ significantly deviates from the δ_crystal_, which measures less than 1 nm, as illustrated in Figure S7 (Supporting Information). The differences between these values could introduce errors in the determination of the GB diffusivity (*D*_gb_)^[^ ^4^, ^5^, ^6^, ^11^, ^21^, ^22^, ^61^ ^]^ and the space charge potential (Φ_0_) of GBs,^[^ ^73^ ^]^ particularly when the commonly used estimation value of δ ≈1 nm is applied. In this study, for the sample annealed at 350 °C, the *D*_gb_ was determined to be 2.5 × 10^−11^ cm^2^ s^−1^, based on the measured δ_chem_ of 11.10 nm, as presented in Figure 4. This result is an order of magnitude lower than the 2.8 × 10^−10^ cm^2^ s^−1^ estimated using the assumed δ_crystal_ of 1 nm. It is also noteworthy that the pseudocubic unit cell of LSCF is around 3.88 Å, nearly 4 Å. Thus, a boundary width of ≈8 – 11 nm implies a compositional change across roughly 20 – 28 unit cells.

Finally, the shapes of the elemental distribution profiles across the GBs obtained through APT (Figures 4 and 6; Figure S4, Supporting Information) and EELS (Figure 7) have confirmed that the distribution of $V_{O}^{\cdot \cdot }$ and cations cannot simply be described as step functions at the core (abrupt core model in the case for metals).^[^ ^31^, ^37^ ^]^ Instead, it is more appropriately modeled as an exponential distribution or a combination of both. Parras and De Souza^[^ ^7^ ^]^ proposed that the cation‐vacancy distribution can be modeled using exponential terms expressed as $\exp\left(-\frac{2e\Phi_{0}}{k_{B}T}\right)\exp\left(-\frac{\Delta \mu }{k_{B}T}\right)$, where Δμ is the chemical potential. However, it is important to consider that the distributions may also be influenced by the presence of impurities. This study further enhances our understanding of *D*_gb_ quantification that the CML does not contribute equally to the oxygen diffusion rate. The *D*_gb_ represents the average diffusivity across the CML and is expected to vary following the cation‐vacancy distribution within the CML. Once again, owing to the accrual of impurities at GB, the regions in close proximity to the core might not exert the most pronounced influence.

## 3 Conclusion

The evolution of CMLs and the chemical composition of GBs are pivotal considerations in understanding and optimizing electrode performance and stability. In this study, the GB transport of LSCF6428 was investigated at both single and multi‐grain scales using a range of comprehensive characterization techniques. The polycrystalline dense LSCF ceramics were prepared through sintering at 1250 °C. The oxygen transport properties were investigated through isotopic exchange under dry oxygen conditions with a pO_2_ of 200 mbar at 350 and 500 °C, respectively. The oxygen diffusivity was found to be significantly enhanced along the GBs compared to that in the bulk at 350 °C under the Harrison type‐B diffusion regime. The D* was measured as 2.5 × 10^−14^ cm^2^ s^−1^. The *D*_gb_ was determined to be 2.8 × 10^−10^ cm^2^ s^−1^ assuming a δ_crystal_ of 1 nm, and 2.5 × 10^−11^ cm^2^ s^−1^ cm^2^/s using a chemically measured δ_chem_ of 11.10 nm by APT. APT analysis on individual GBs further revealed that the enhancement is attributable to the accumulation of $V_{O}^{\cdot \cdot }$, the defects responsible for oxygen diffusion at the GB region. The observation of faster GB transport for oxygen diminished at 500 °C despite a similar level of oxygen deficiency found on one of the GB. This is a result of an increase in oxygen bulk diffusivity, transitioning from a Harrison type‐B to a type‐A scenario.

Some general trends can be discerned regarding cation distributions across GBs through APT analysis. An observed ppm level of impurities, specifically Na, could potentially impact the space charge potential, consequently influencing the GB chemistry evolution. In one of the GB of the sample annealed at 350 °C, a counterintuitive deficiency in the [$V_{O}^{\cdot \cdot }$] was noted at the GB core compared to the adjacent CMLs. At the core, a 2.6(7)% oxygen deficiency was noted, in contrast to the 4.3(7)% at the CMLs, most likely attributed to the presence of Na. The enrichment of Sr, ≈7% was found, which can be explained by the charge compensation of the positively charged $V_{O}^{\cdot \cdot }$ and the negatively charged acceptor dopant, $Sr_{La}^{\prime }$. The other A‐site cation, La, was deficient at the GB region in a similar amount, complementing the enrichment of Sr. In addition, the B‐site cations, Co and Fe, also tend to segregate toward GBs forming complexions with Sr and/or Na, driven by a thermodynamic force simulated using DFT. It is noteworthy that, despite the trends, the varying levels of enrichment and deficiency for each cation emphasize the distinct chemistry of each GB. Furthermore, it is noteworthy that there is a difference between the crystallographic δ (≤1 nm) and the chemically defined δ (a few nm). This distinction could introduce inaccuracies in the calculation of *D*_gb_ and the Φ_0_ of GBs. The scientific findings advances our understanding of the cation‐vacancy distribution at the GB, and the determination of the *D*_gb_. These results are anticipated to catalyze new research initiatives on GB engineering and contribute to advancements in material design, performance, and the durability of electrodes.

## 4 Experimental Section

The La_0.6_Sr_0.4_Co_0.2_Fe_0.8_O_3‐δ_ (LSCF6428) powders (Praxair Inc, US, LOT: 03‐P6171DM) were uniaxially pressed at a load of 2 tons in a 13 mm diameter die. Subsequently, the green pellets were sintered in a muffle furnace under static laboratory air at 1250 °C for 8 h to prepare dense ceramic pellets with > 99% of the theoretical density. The sintered pellets were further ground using successive grades of SiC papers (Struers Ltd., UK) with grits of 400, 600, 800, and 1200, and then polished with water‐based diamond suspensions (Struers Ltd., UK) of 6, 3, 1, and ¼ µm sequentially to reduce errors arising from surface roughness.

The oxygen transport properties were investigated through oxygen isotope exchange depth profiling (IEDP) techniques coupled with both secondary ion mass spectrometry (SIMS). The oxygen isotopic exchange was conducted in a dry oxygen environment (≈1 vppm H_2_O), with an oxygen partial pressure (pO_2_) of 200 mbar at both 350 and 500 °C, respectively. The exchange process consists of two stages: a pre‐anneal in ^16^O_2_ (BOC Ltd, N5.0, corresponding to the “dry” conditions) for 24 h to eliminate any chemical gradient of oxygen within the sample, followed by an exchange‐anneal in ^18^O_2_ (CK Isotopes Ltd, with an enrichment measured to be 88%) for 3 h. The ^18^O diffusion profiles were measured using time‐of‐flight SIMS (TOF‐SIMS 5, IONTOF GmbH, Germany) in the depth profiling mode, and the crater depth after SIMS analysis was measured using a 3D optical interferometer (NewView 200, Zygo Corporation, US). Crank's solution to the diffusion equation in a semi‐infinite medium,^[^ ^51^ ^]^ as described in Equation 5, was applied to fit the diffusion profiles and obtain the oxygen tracer diffusion coefficient, D*, which characterizes bulk diffusivity, as well as the surface exchange coefficient, *k*, which characterizes surface exchange kinetics.

(5) $$\begin{array}{ccc} C^{\prime }\;\left(x,t\right) & = & \frac{C\left(x,t\right)-C_{bg}}{C_{g}-C_{bg}}\;=\;erfc\left[\frac{x}{2\sqrt{D^{*}t}}\right]\\ & & -\left[\exp\left(\frac{kx}{D^{*}}+\frac{k^{2}t}{D^{*}}\right)\times erfc\left(\frac{x}{2\sqrt{D^{*}t}}+k\sqrt{\frac{t}{D^{*}}}\right)\right] \end{array}$$

where *C*′(*x*, *t*) is the normalised isotopic fraction, *C*(*x*, *t*) is the ratio of ^18^O signal intensity to the total intensity of (^16^O + ^18^O) measured by SIMS, *C_bg_* is the natural isotopic background of ^18^O, *C_g_* is the isotopic fraction of the annealing gas, *x* is distance from the sample surface, *t* is the ^18^O isotope exchange anneal time. A non‐linear least squares method was used for the fitting with the in house MATlab app TraceX.^[^ ^77^ ^]^

The GB chemistry of the annealed samples at 350 and 500 °C, respectively, for 27 h (24 h of pre‐annealing plus 3 h of exchange annealing) was further studied using atom probe tomography (APT) and transmission electron microscopy (TEM). The needle‐shaped APT specimens with an apex less than 100 nm, illustrated in Figure S8 (Supporting Information), were prepared using focused ion beam (FIB) milling and subsequent lift‐out. This process was carried out with a focused ion beam scanning electron microscope (FIB‐SEM, Thermofisher Scientific Helios 5, UK) following the procedure detailed in the previous literature.^[^ ^78^ ^]^ The APT measurements were conducted at 55 K in laser pulsing mode using a laser energy of 10 pJ, a pulse frequency of 200 kHz, and a detection rate of 1% (Cameca LEAP 5000 XR, France). The acquired data were analyzed using the Cameca integrated visualization and analysis software (IVAS) within the AP suite 6.1 toolkit. Peak identification was performed using known isotopic ratios in the spectra, with standard IVAS background correction applied. Thin electron‐transparent TEM lamellae were also prepared using the FIB‐SEM through lift‐out the region of interested, followed by thinning with the Ga ion milling. The TEM analysis was performed with a JEM‐2010F TEM/STEM (JEOL Ltd, Japan) operated at 200 kV acceleration voltage and equipped with a Gatan GIF electron energy‐loss spectrometer (EELS).

The surface chemistry of the LSCF6428 was analyzed using X‐ray photoelectron spectroscopy (XPS) using a high‐throughput X‐ray photoelectron spectrometer (K‐Alpha+, Thermo Fisher Scientific, US) equipped with a monochromated Al Kα radiation source (*h*ν = 1486.6 eV). Survey and core‐level spectra were collected with pass energies of 200 and 20 eV, respectively. The spectra were processed by subtracting a Shirley‐type background,^[^ ^71^ ^]^ and the peaks were fitted using a Gaussian‐Lorentzian line shape. The surface chemistry and morphology were further studied using scanning electron microscopy (SEM, Carl Zeiss Ltd, Germany) equipped with a LEO Gemini 1525 field emission gun (FEG).

The cation‐vacancy distribution and reactions with Na impurities at the GBs were also predicted using density functional theory (DFT) energetics. The energies of structures within the La‐Sr‐Fe‐Co‐O‐Na phase diagram were taken from the Materials Project Database.^[^ ^67^ ^]^ Phase diagrams were created using the pymatgen package.^[^ ^79^, ^80^ ^]^ The energy of a single unit cell structure of La_3_Sr_2_Fe_4_CoO_15_ (La_0.6_Sr_0.4_Fe_0.8_Co_0.2_O_3_) was calculated with DFT+U, using the same input parameters and energy corrections as used in the Materials Project.^[^ ^67^ ^]^ The structure was fully optimized in the ferromagnetic state in the VASP package^[^ ^81^ ^]^ using PAW pseudopotentials.^[^ ^82^ ^]^ The structure was optimized until the force on any atom fell below 0.01 eV atom^−1^, using a plane wave cut off of 520 eV. Hubbard *U* parameters were added to Fe and Co to treat electron correlation, using the rotationally invariant formalization of Dudarev et al.^[^ ^83^, ^84^ ^]^ Values of *U*_eff_ = 5.3 and 3.32 were added to the *d* states of Fe and Co, respectively, based on the previous work of Wang et al.^[^ ^85^ ^]^ The impact of temperature (T) and pO_2_ on the oxygen chemical potential (μ_*O*_) was included in the reaction energy calculations by assuming that O_2_ behaves as an ideal gas and follows Equation 6:

(6) $$\mu_{O_{2}}\;\left(T,\;pO_{2}\right)=\;\mu_{O_{2}}\left(T,\;p_{0}\right)+k_{B}Tln\frac{pO_{2}}{p_{0}}\;$$

where p_0_ is a reference partial pressure, and k_B_ is Boltzmann's constant.^[^ ^79^ ^]^ Values for the reference chemical potential were taken from the JANAF thermochemical database.^[^ ^86^ ^]^ All reactions were calculated at an oxygen partial pressure of pO_2_ = 200 mbar.

## Conflict of Interest

The authors declare no conflict of interest.

## Supporting information



Supporting Information

## Data Availability

The data that support the findings of this study are available from the corresponding author upon reasonable request.
