# Supplementary material for: Revealing Local Grain Boundary Chemistry and Correlating it with Local Mass Transport in Mixed‐Conducting Perovskite Electrodes
Source: Small. 2024 Oct 4;20(50):2404702. doi: 10.1002/smll.202404702 (PMC11636158; doi:10.1002/smll.202404702)
Supplement: Supplementary file 1 — Supporting Information [file SMLL-20-2404702-s001.docx]

**Supplementary Information**

**Revealing local grain boundary chemistry and correlating it with local mass transport in mixed-conducting perovskite electrodes**

Zijie Sha^a^, James O. Douglas^a^, Lluís Yedra^b,c^, Ieuan D. Seymour^d^, Sònia Estradé^b,c^, Francesca Peiró^b.c^, Stephen J. Skinner^a^, and John A. Kilner^a^

^a^Department of Materials, Imperial College London, London, SW7 2AZ, United Kingdom.

^b^Laboratory of Electron Nanoscopies (LENS), Department of Electronics and Biomedical Engineering, Universitat de Barcelona, c/ Marti Franquès 1, 08028 Barcelona, Spain.

^c^Institute of Nanoscience and Nanotechnology (IN2UB), Universitat de Barcelona, Diagonal 645, Barcelona, 08028, Spain

^d^Advanced Centre for Energy and Sustainability (ACES), Department of Chemistry, University of Aberdeen, Aberdeen AB24 3UE, Scotland, United Kingdom.

J.Kilner@imperial.ac.uk

**Section S-1:**.


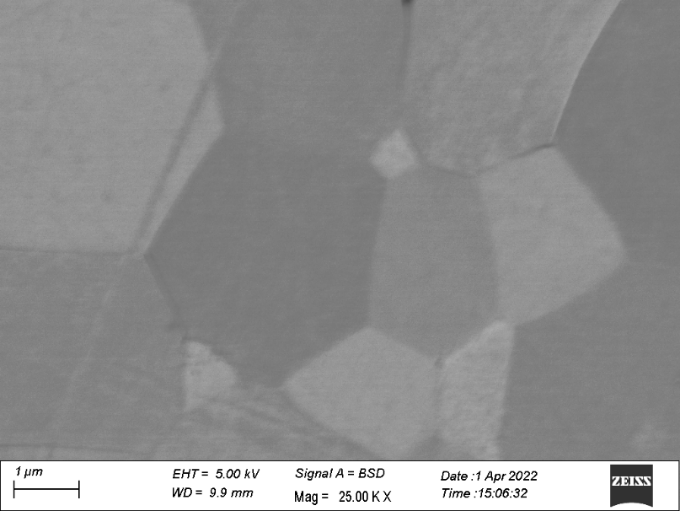

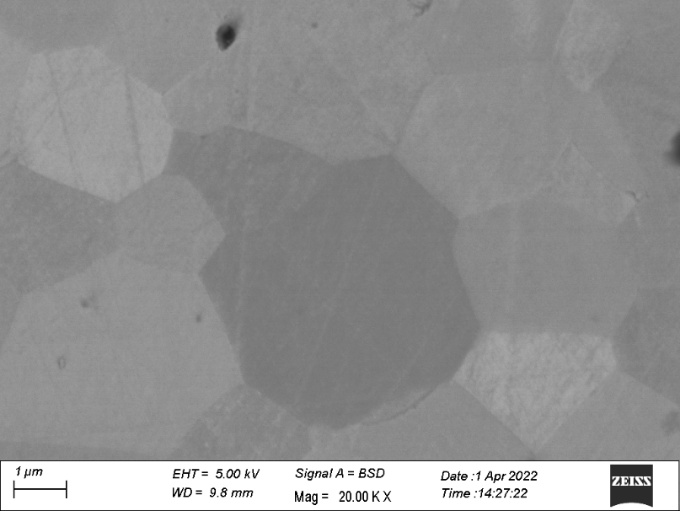


**(a)**

**(b)**

**(c)**

**(d)**


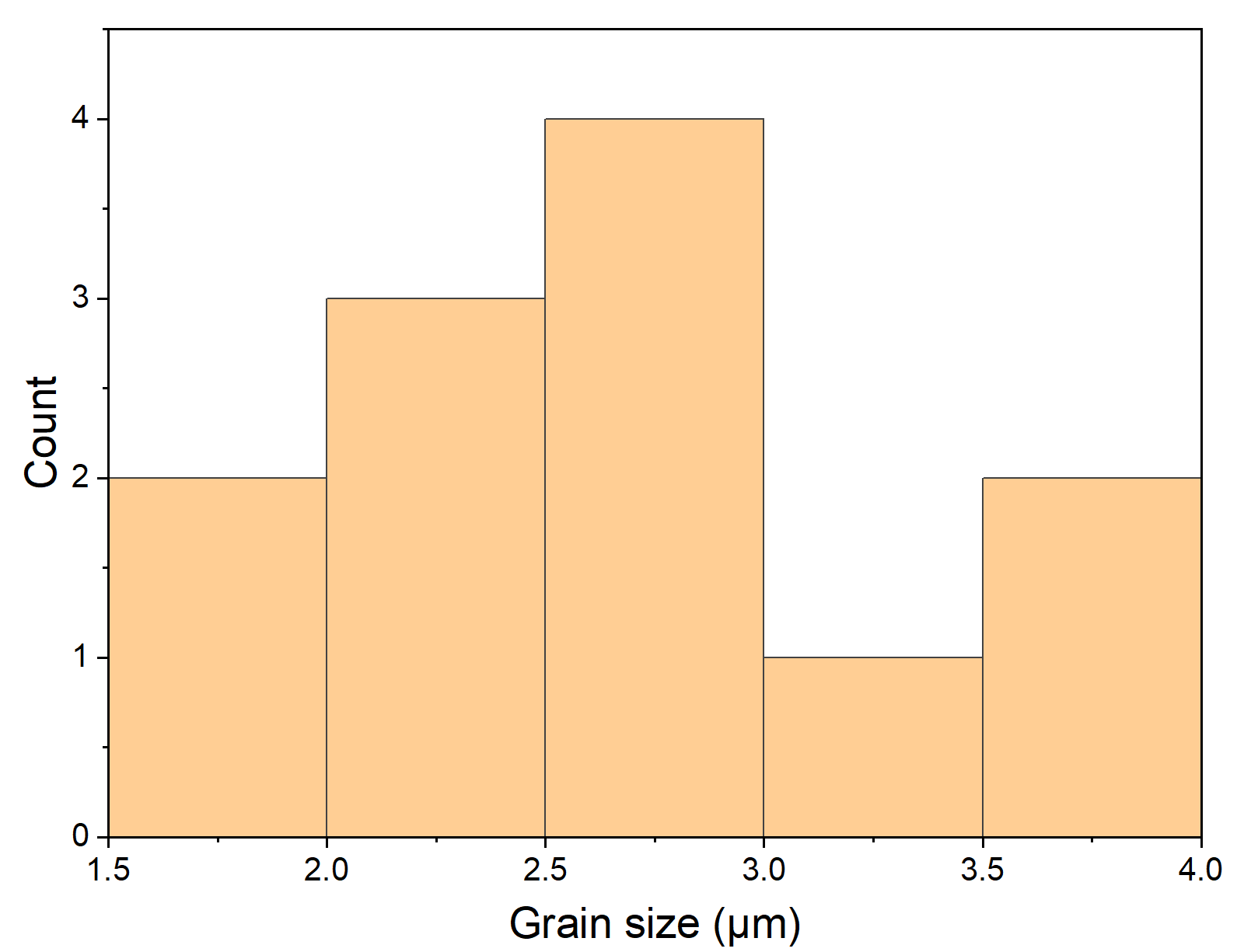

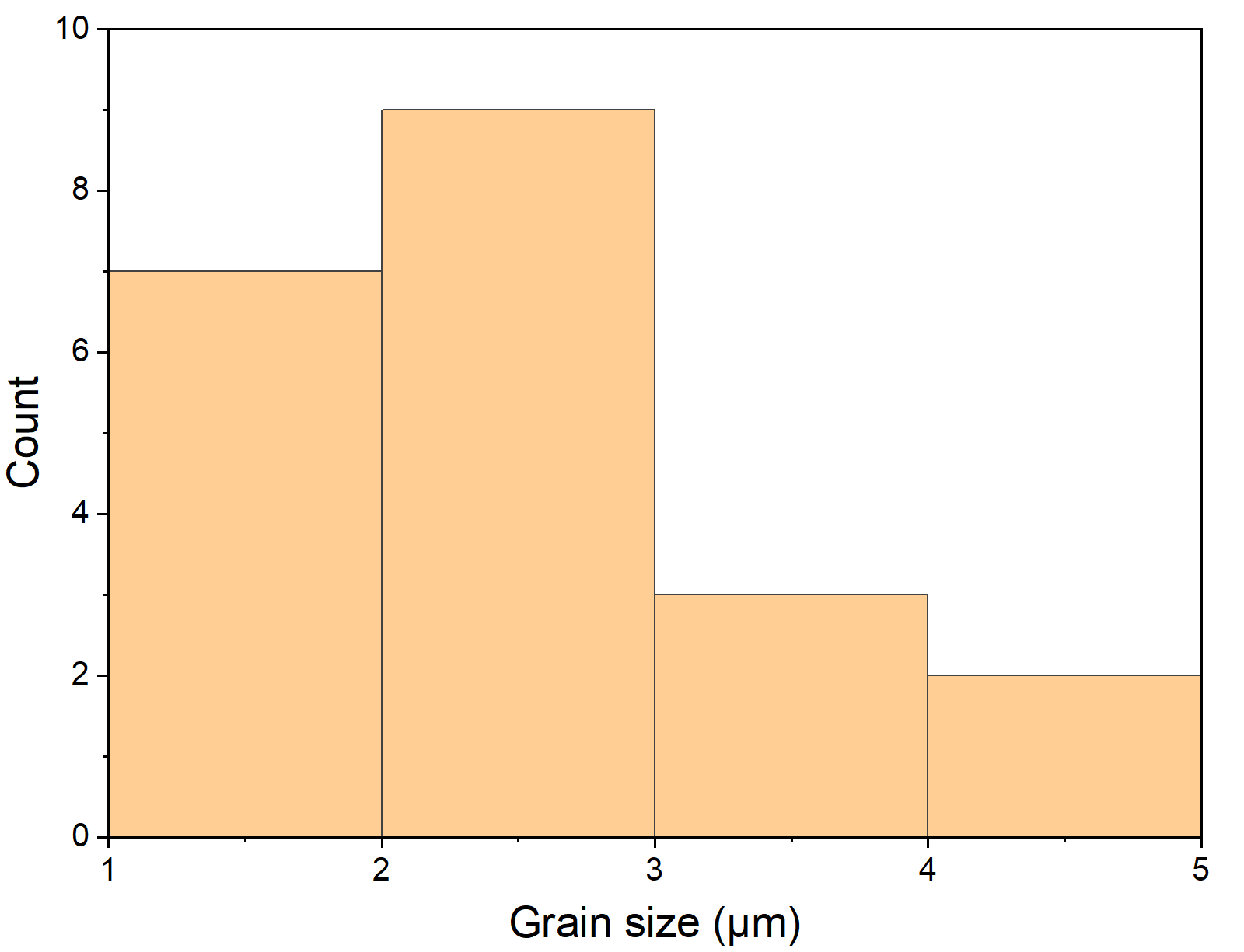


**Figure S1.** (a – b) SEM images of the LSCF6428 sample annealed at (a) 350°C, and (b) 500°C. (c – d) Grain size analysis of the LSCF6428 sample annealed at (c) 350°C, and (d) 500°C.

**Section S-2:**

The total impurity level of Na in the LSCF sample, *f*_Na_, can be estimated from the APT results, assuming a cubic grain with a size of *L =* 2.5(1) μm, as shown in Figure S2, and an impurity-enriched GB region with a thickness of *l*^1^:

$$f_{Na}=\frac{\frac{6lL^{2}c_{Na}^{gb}}{2}+c_{i}^{bulk}L^{3}}{L^{3}}=\frac{3lC_{Na}^{gb}}{L}+C_{i}^{bulk}$$

where $C_{Na}^{gb}$ and $C_{Na}^{bulk}$ represent the concentration of Na in the GB and the bulk, respectively, as obtained from the values displayed in Figure 4b in the main text, with *l* approximately equal to 11.1 nm.

The standard error, *S*, in the 1D elemental profiles across a GB, obtained by APT was calculated as:

$$S= \sqrt{\frac{C_{i}(1-C_{i})}{n_{t}}}$$

where *C*_i_ represents the concentration in atomic fraction of a solute in the sample, defined as:

$$C_{i}=\frac{n_{i}}{n_{t}}$$

where *n*_i_ is the number of ions of solute *i* in the sample and *n*_t_ is the total number of ions in the sample.

**Section S-3:**


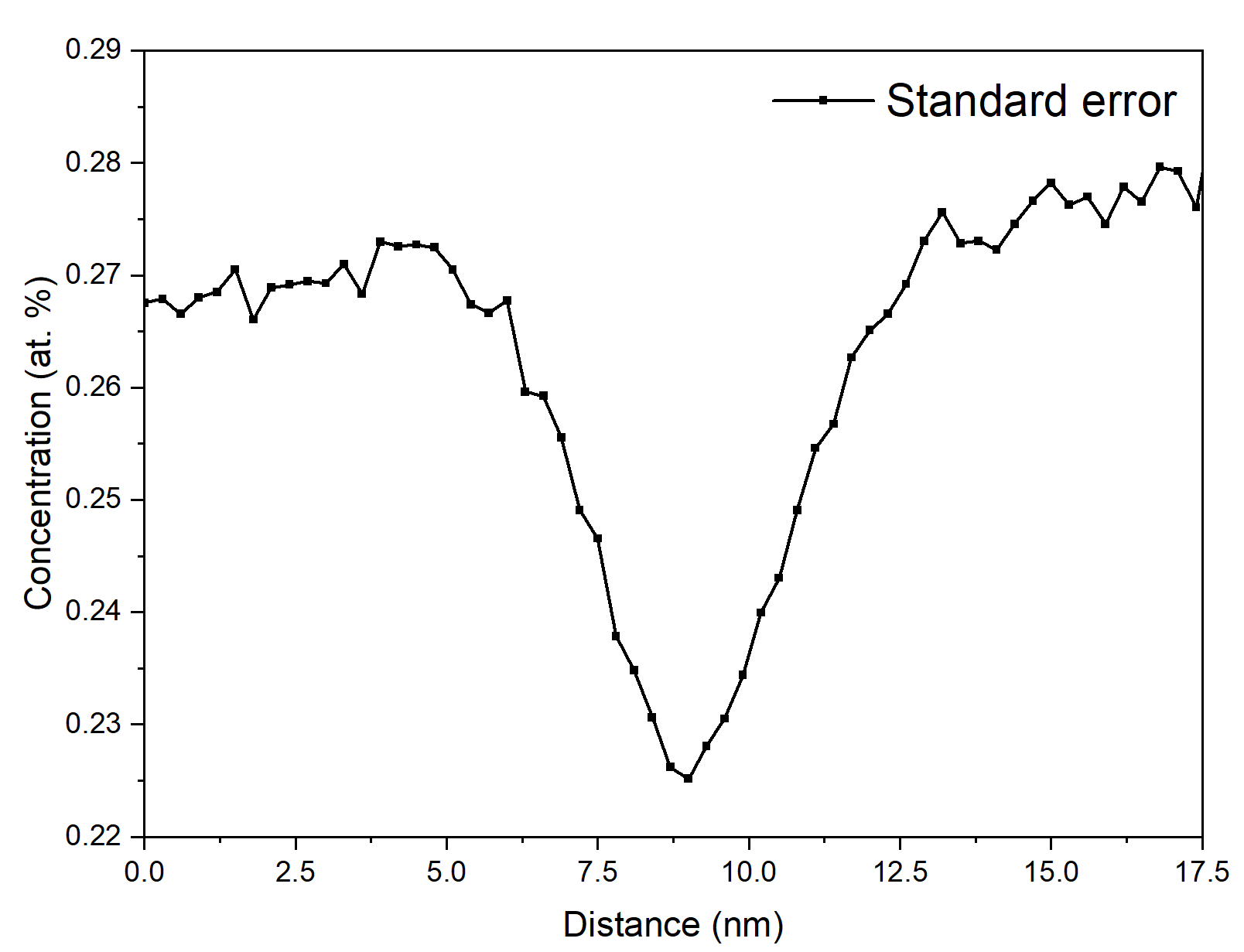

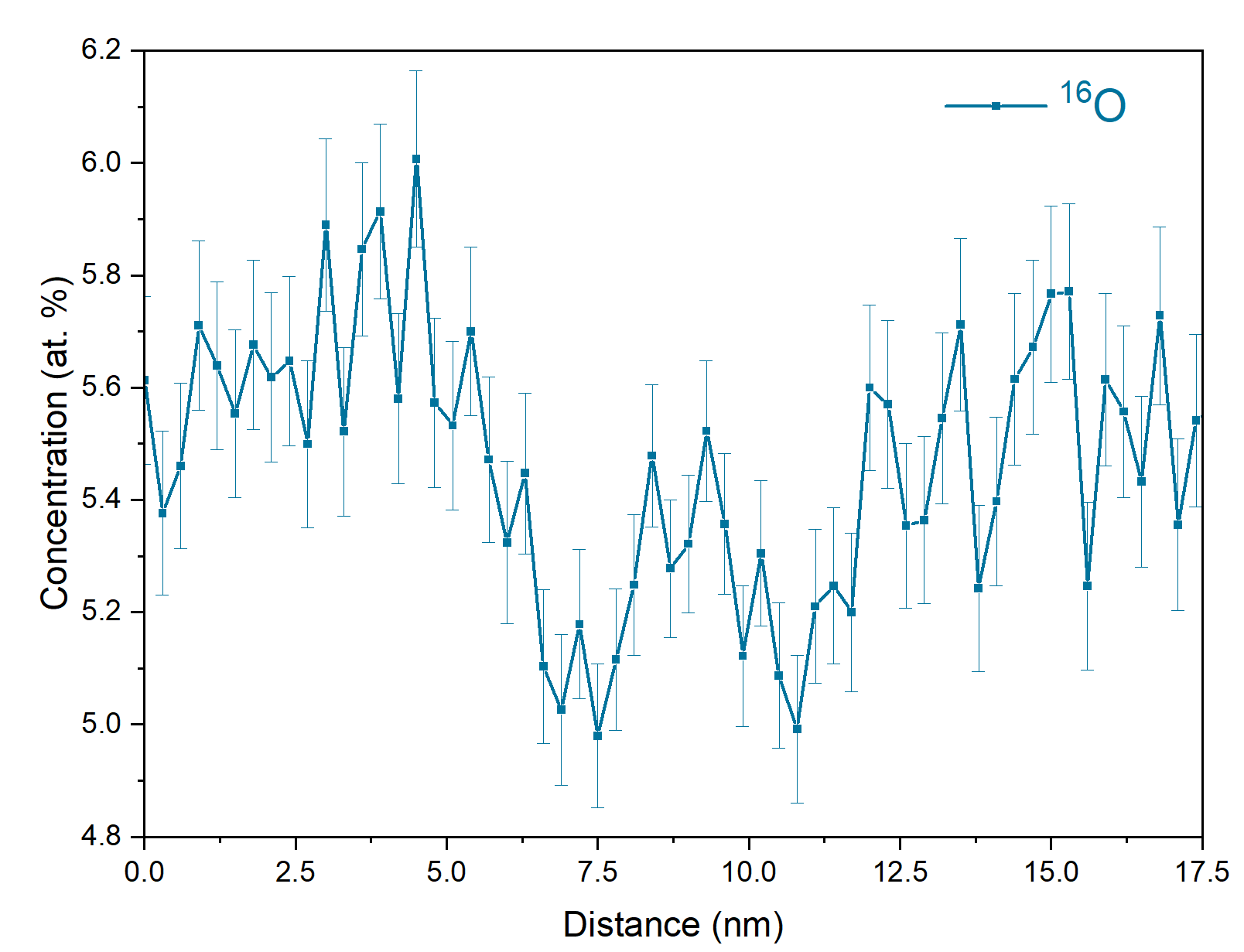


**(a)**

**(b)**

**Figure S2.** (a) The plot of the standard errors of the O signal displayed in Figure 4c in the main text. (b) Elemental distribution profiles of ^16^O across the GB1, as shown in Figure 4a in the main text, obtained from the LSCF6428 sample annealed at 350°C. The standard error was calculated based on the ion counts.


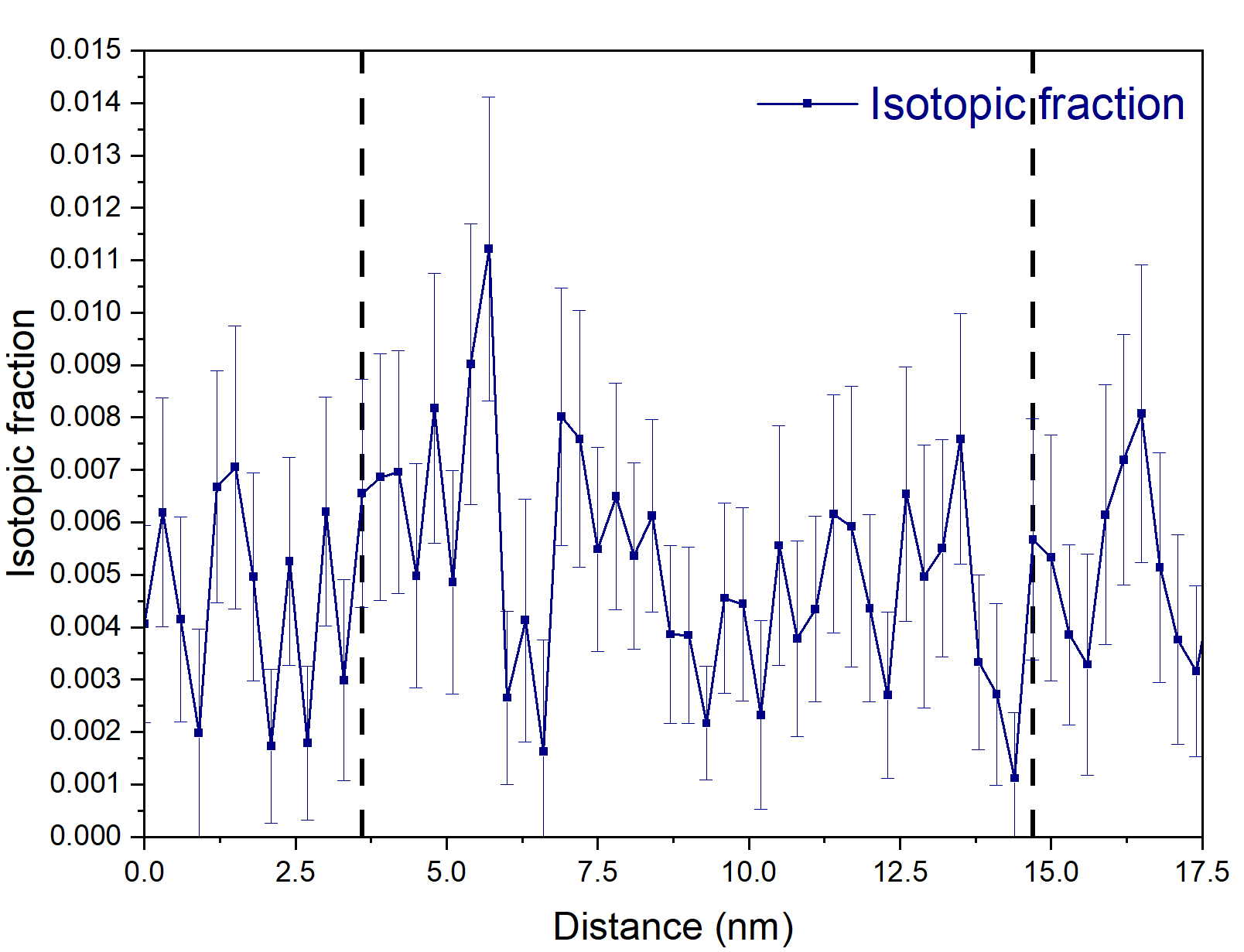


**Figure S3.** The isotopic fraction of $\frac{[Mass 18]}{\left[ Mass 18 \right]+[Mass 16]}$ across the GB1, as presented in Figure 4a in the main text, of the LSCF6428 sample annealed at 350°C.


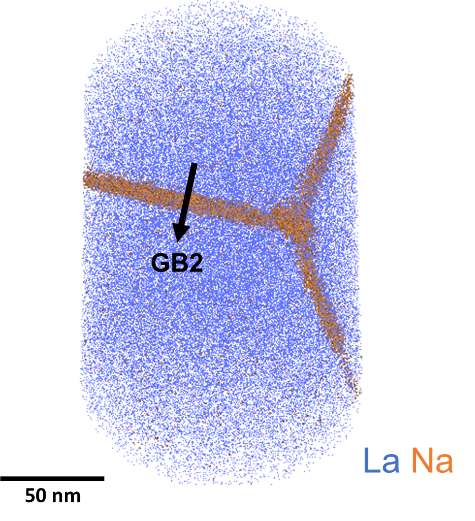

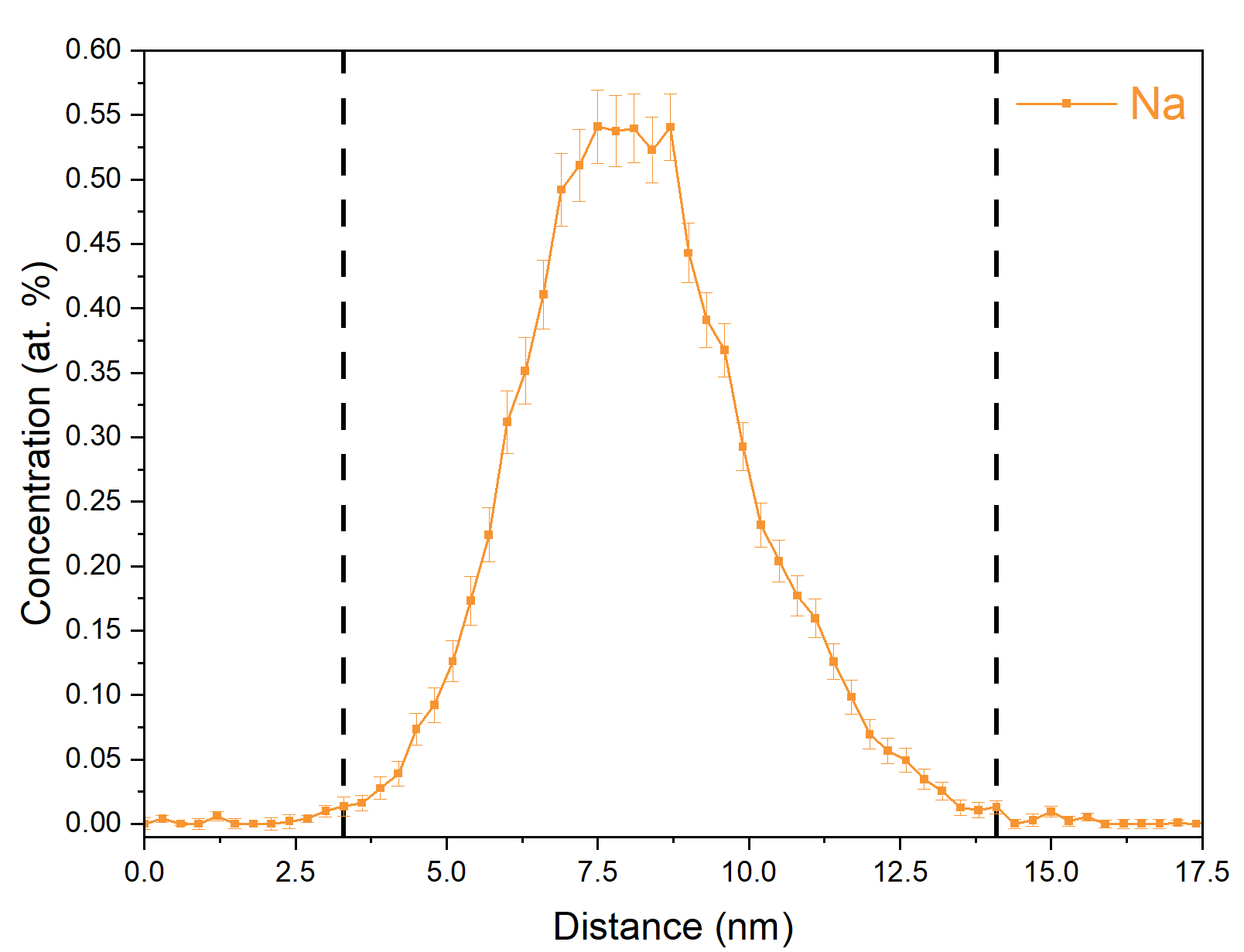


**(a)**

**(b)**

**(c)**

**(d)**

**(e)**

**(f)**


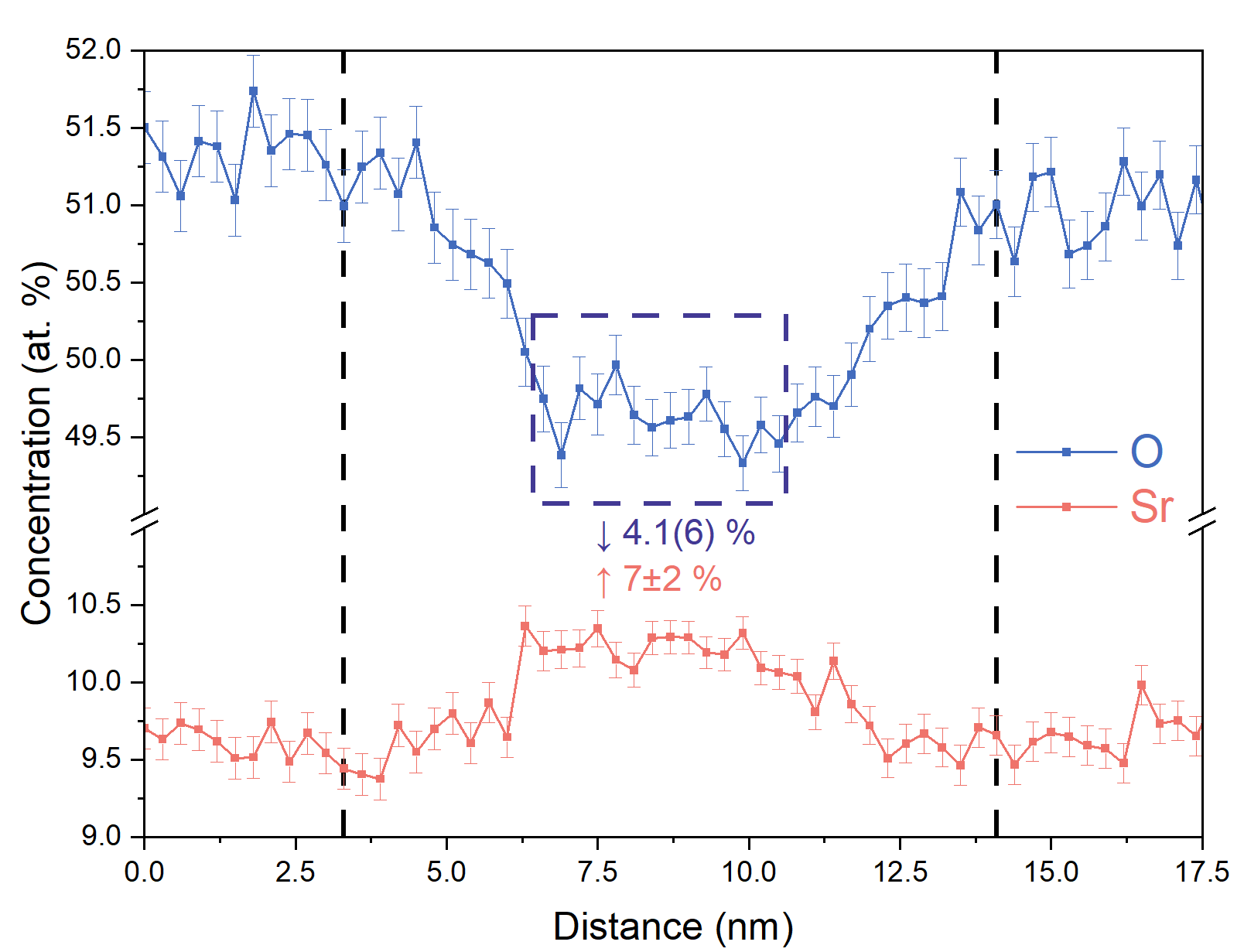

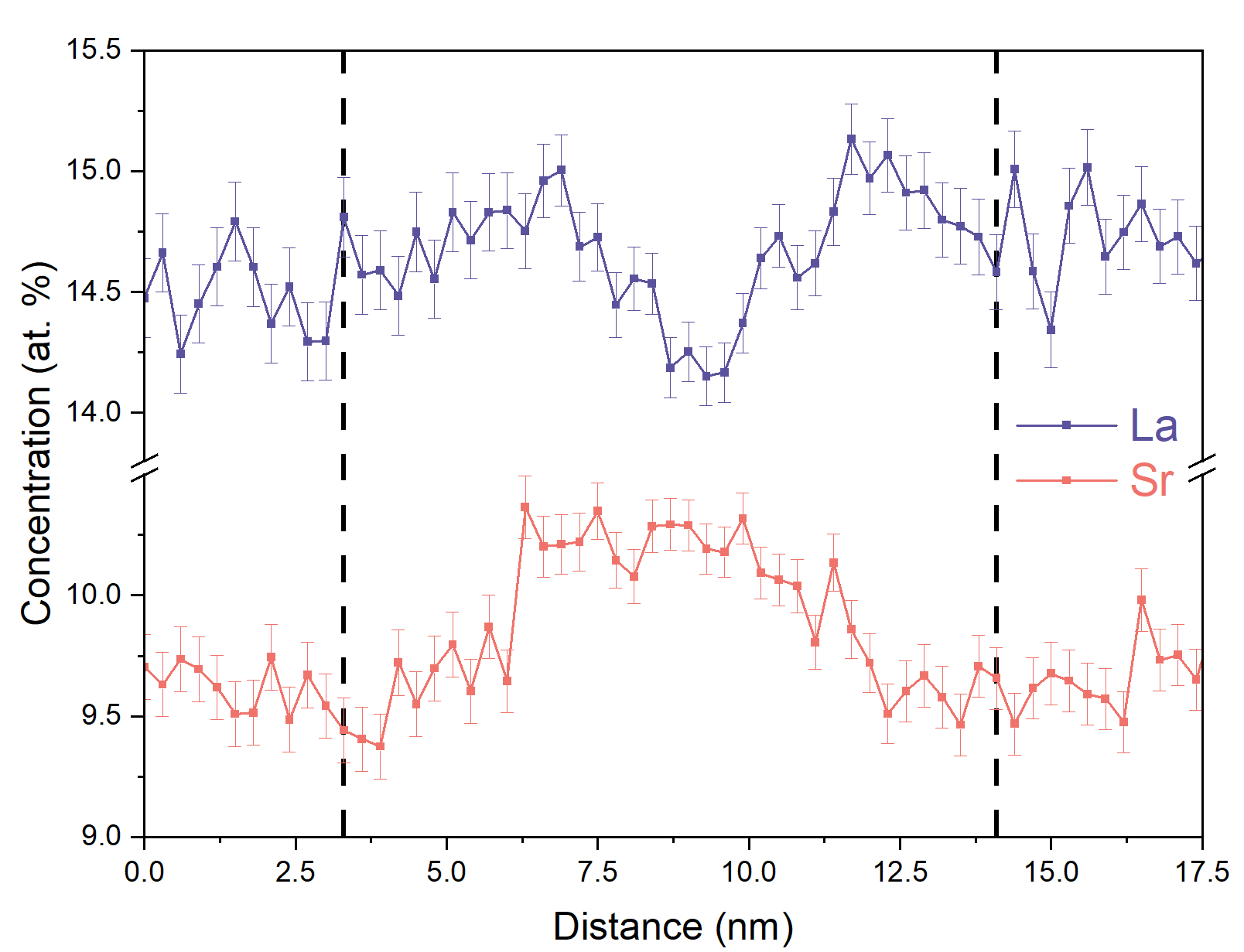


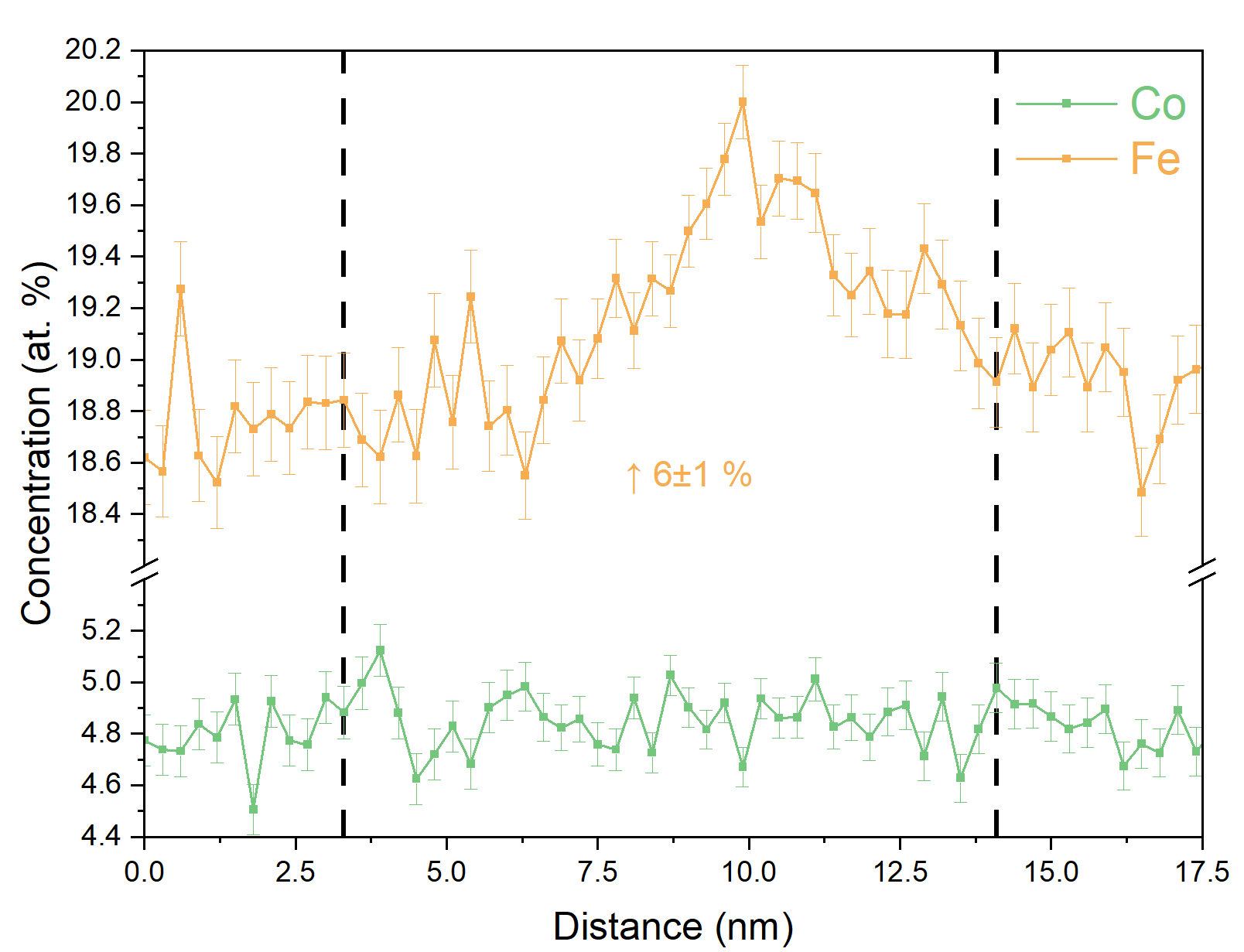

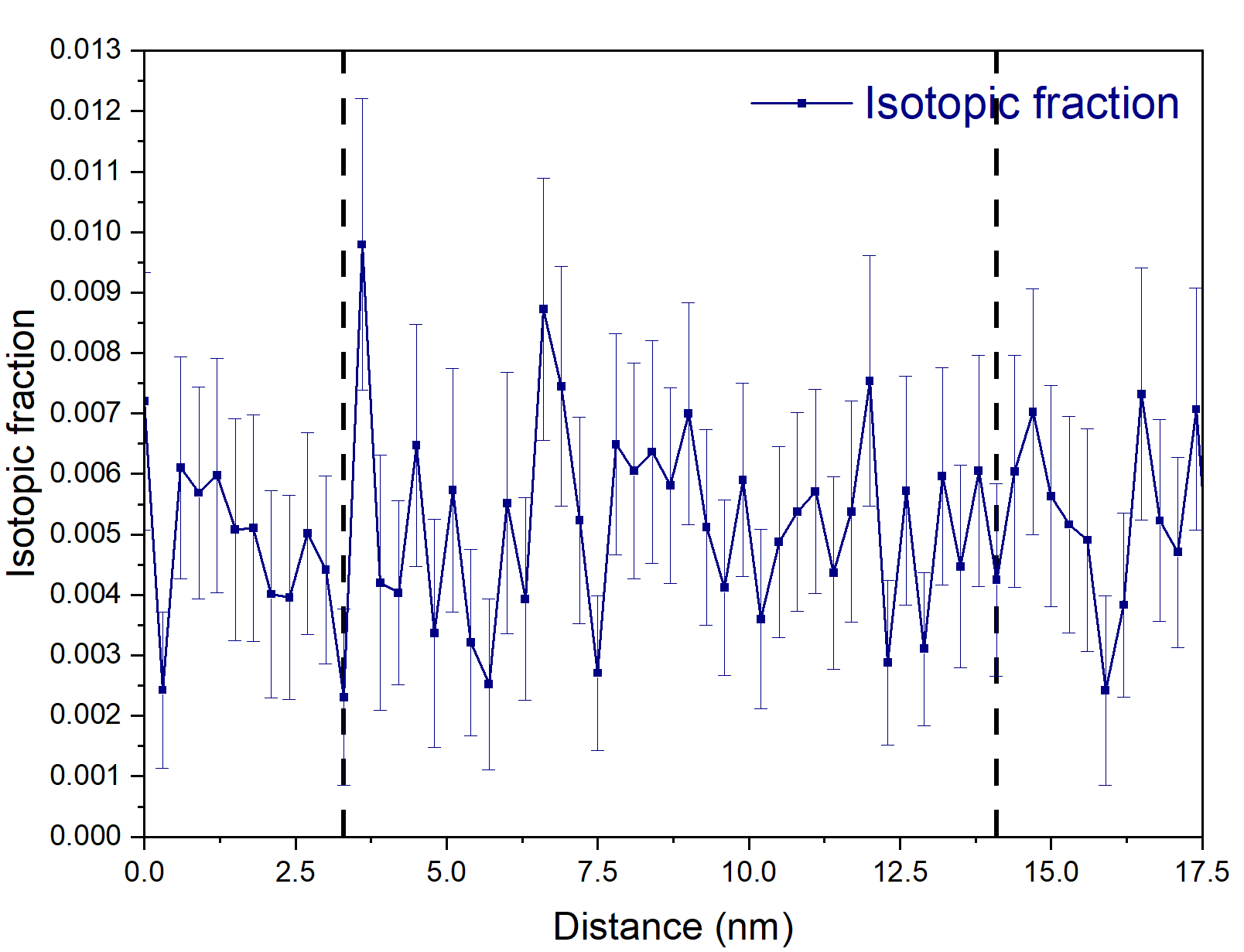


**Figure S4.** (a) APT reconstruction of the LSCF6428 sample annealed at 350°C. The interaction volume is 73.5 × 123.7 × 18.0 nm^3^. The blue dots represent La matrix atoms, while the orange spheres represent Na, indicating the presence of the GB region. (b – f) Elemental distribution profiles of (b) Na; (c) O versus Sr; (d) La versus Sr; (e) Co versus Fe; and (f) isotopic fraction ($\frac{[Mass 18]}{\left[ Mass 18 \right]+[Mass 16]})$ across GB1, as highlighted in Figure S5a.

**Section S-4:**

The full La-Sr-Fe-Co-O phase diagram was generated to predict the decomposition products of oxygen deficient La_0.6_Sr_0.4_Fe_0.8_Co_0.2_O_3-δ_ systems. A single unit cell structure of La_0.6_Sr_0.4_Fe_0.8_Co_0.2_O_3_ was added to the phase diagram and after full geometry optimisation, was found to be +18 meV/atom above the convex hull. As thermal energy is ~25 meV/atom at 300 K, this configuration may be stabilised at elevated temperatures by configuration entropy. For subsequent calculations, the energy of the La_0.6_Sr_0.4_Fe_0.8_Co_0.2_O_3_ phase was lowered to sit exactly on the energy hull.

Using the bulk energetics of the La-Sr-Fe-Co-O phase diagram, the decomposition products of a hypothetical oxygen deficient La_0.6_Sr_0.4_Fe_0.8_Co_0.2_O_2.999_ phase were predicted at four temperatures: 0, 350, 500 and 1250°C to explore the impact of sintering and thermal annealing. The impact of temperature was included via the change in the chemical potential of O_2_ gas using the ideal gas formula. The following decomposition reactions were predicted:

La_0.6_Sr_0.4_Fe_0.8_Co_0.2_O_2.999_ (0°C) 🡪

0.9767 La_0.6_Sr_0.4_Fe_0.8_Co_0.2_O_3_ + 0.014 LaFeO_3_ + 0.00033 Sr_18_Co_14_O_45_ + 0.0033 SrFeO_3_ +0.00067 Fe_2_O_3_

La_0.6_Sr_0.4_Fe_0.8_Co_0.2_O_2.999_ (350°C) 🡪

0.9867 La_0.6_Sr_0.4_Fe_0.8_Co_0.2_O_3_ + 0.0080 LaFeO_3_ + 0.00067 Sr_5_Co_4_O_12_ + 0.002 SrFeO_3_ +0.00033 Fe_2_O_3_

La_0.6_Sr_0.4_Fe_0.8_Co_0.2_O_2.999_ (500°C) 🡪

0.9925 La_0.6_Sr_0.4_Fe_0.8_Co_0.2_O_3_ + 0.0045 LaFeO_3_ + 0.00038 Sr_5_Co_4_O_12_ + 0.00025 Sr_2_FeO_4_ + 0.000625 SrFe_2_O_4_

La_0.6_Sr_0.4_Fe_0.8_Co_0.2_O_2.999_ (1250°C) 🡪

0.9964 La_0.6_Sr_0.4_Fe_0.8_Co_0.2_O_3_ + 0.0020 LaFeO_3_ + 0.00014 SrLaCoO_4_ + 0.00043Sr_3_Fe_2_O_6_ + 0.00057 CoO

For all temperatures studied, decomposition of the La_0.6_Sr_0.4_Fe_0.8_Co_0.2_O_2.999_ phase was predicted to lead to a Co, Fe and Sr rich oxides, in addition to the perovskite LaFeO_3_ phase. The Sr_5_Co_4_O_12_, Sr_5_Co_4_O_12_, Sr_2_FeO_4_, SrFe_2_O_4_, SrLaCoO_4_, Sr_3_Fe_2_O_6_ and CoO complexions have a metal to oxygen ratio between X_M_/X_O_ = 3/4 – 1, higher than in the perovskite *AB*O_3_, where X_M_/X_O_ =2/3. If local environments at the GB adopted compositions similar to these complexions, it would lead to a decrease in the oxygen atom fraction, which is what is observed experimentally.

The role of Na on the phase energetics was also explored, Na was found to be present at the grain boundary from APT. The full La-Sr-Fe-Co-O-Na phases diagram was therefore generated. The reaction between 0.001 Na_2_O was considered with the O deficient La_0.6_Sr_0.4_Fe_0.8_Co_0.2_O_2.999_ phases at 0, 350 and 500°C, with the resulting reaction shown below:

La_0.6_Sr_0.4_Fe_0.8_Co_0.2_O_2.999_ + 0.001 Na_2_O (0°C) 🡪

0.886 La_0.6_Sr_0.4_Fe_0.8_Co_0.2_O_3_ + 0.0684 LaFeO_3_ + 0.0012 Sr_18_Co_14_O_45_ + 0.0228 SrFeO_3_ + 0.00067 SrO_2_ +0.002 NaCo_3_O_6_

La_0.6_Sr_0.4_Fe_0.8_Co_0.2_O_2.999_ + 0.001 Na_2_O (350°C) 🡪

0.910 La_0.6_Sr_0.4_Fe_0.8_Co_0.2_O_3_ + 0.054 LaFeO_3_ + 0.003 Sr_5_Co_4_O_12_ + 0.018 SrFeO_3_ + 0.003 O_2_ + 0.002 NaCo_3_O_6_

La_0.6_Sr_0.4_Fe_0.8_Co_0.2_O_2.999_ + 0.001 Na_2_O (500°C) 🡪

0.9825 La_0.6_Sr_0.4_Fe_0.8_Co_0.2_O_3_ + 0.0105 LaFeO_3_ + 0.0035 Sr_2_FeO_4_ + 0.0005 Na_4_Co_7_O_14_

La_0.6_Sr_0.4_Fe_0.8_Co_0.2_O_2.999_ + 0.001 Na_2_O (1250°C) 🡪

0.9875 La_0.6_Sr_0.4_Fe_0.8_Co_0.2_O_3_ + 0.007 LaFeO_3_ + 0.00050 SrLaCoO_4_ + 0.0015 Sr_3_Fe_2_O_6_ + 0.0018 O_2_ + 0.002 NaCoO_2_

At all temperatures it is predicted that the presence of Na_2_O will lead to the formation of a Na-Co-O complexion (NaCo_3_O_6_, Na_4_Co_7_O_14_ or NaCoO_2_). As the temperature increases, Co^4+^ in the Na-Co-O structures is progressively reduced to Co^3+^. This result suggests that there is a preference for Co to preferentially segregate to form complexions with Na. O deficient/Sr-rich environments are also predicted to be present, which also explains the reduced oxygen signal and increased Sr signal seen experimentally.

**Section S-5:**

**
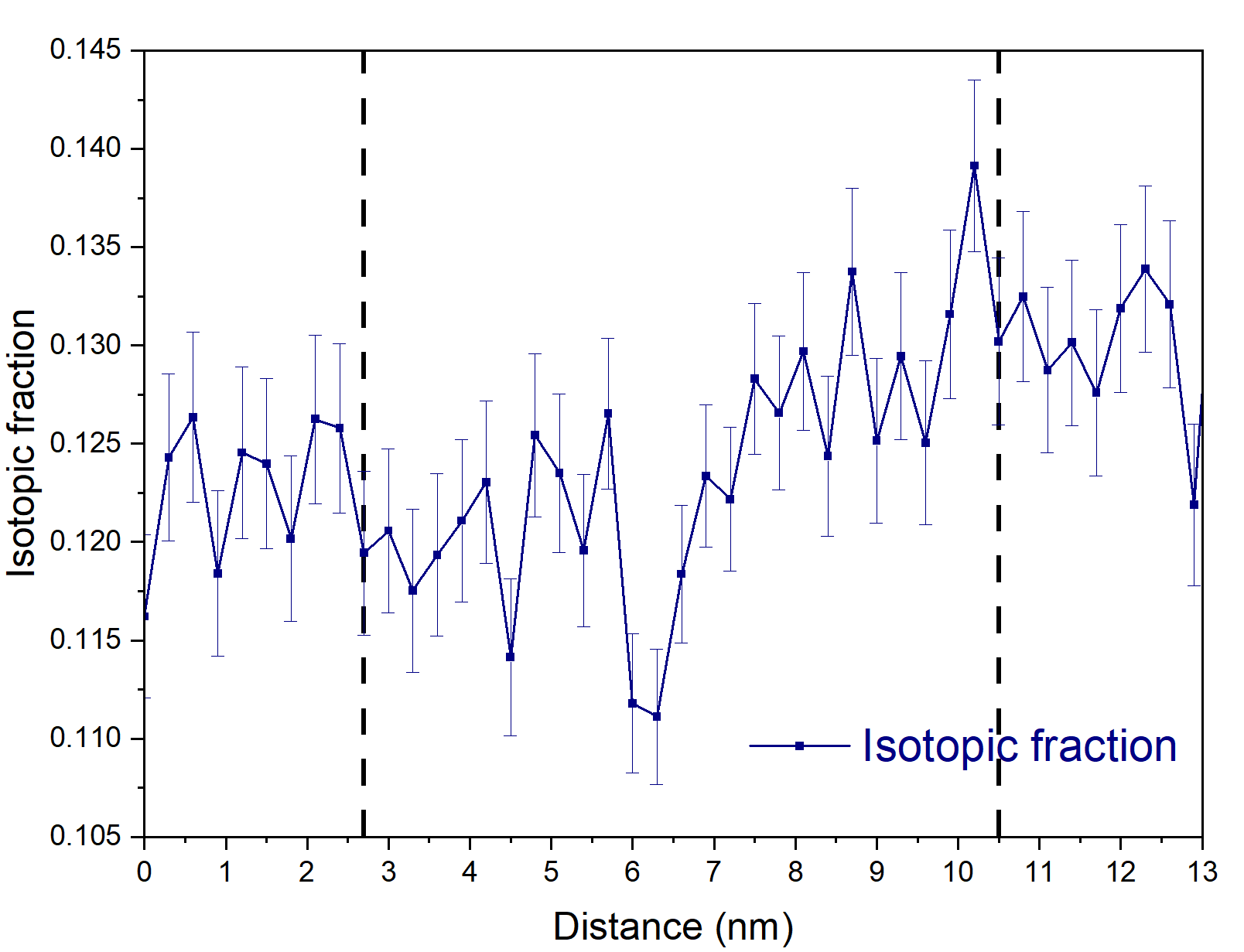
**

**Figure S5.** The isotopic fraction of $\frac{[Mass 18]}{\left[ Mass 18 \right]+[Mass 16]}$ across the GB, as presented in Figure 6a in the main text, of the LSCF6428 sample annealed at 500°C.


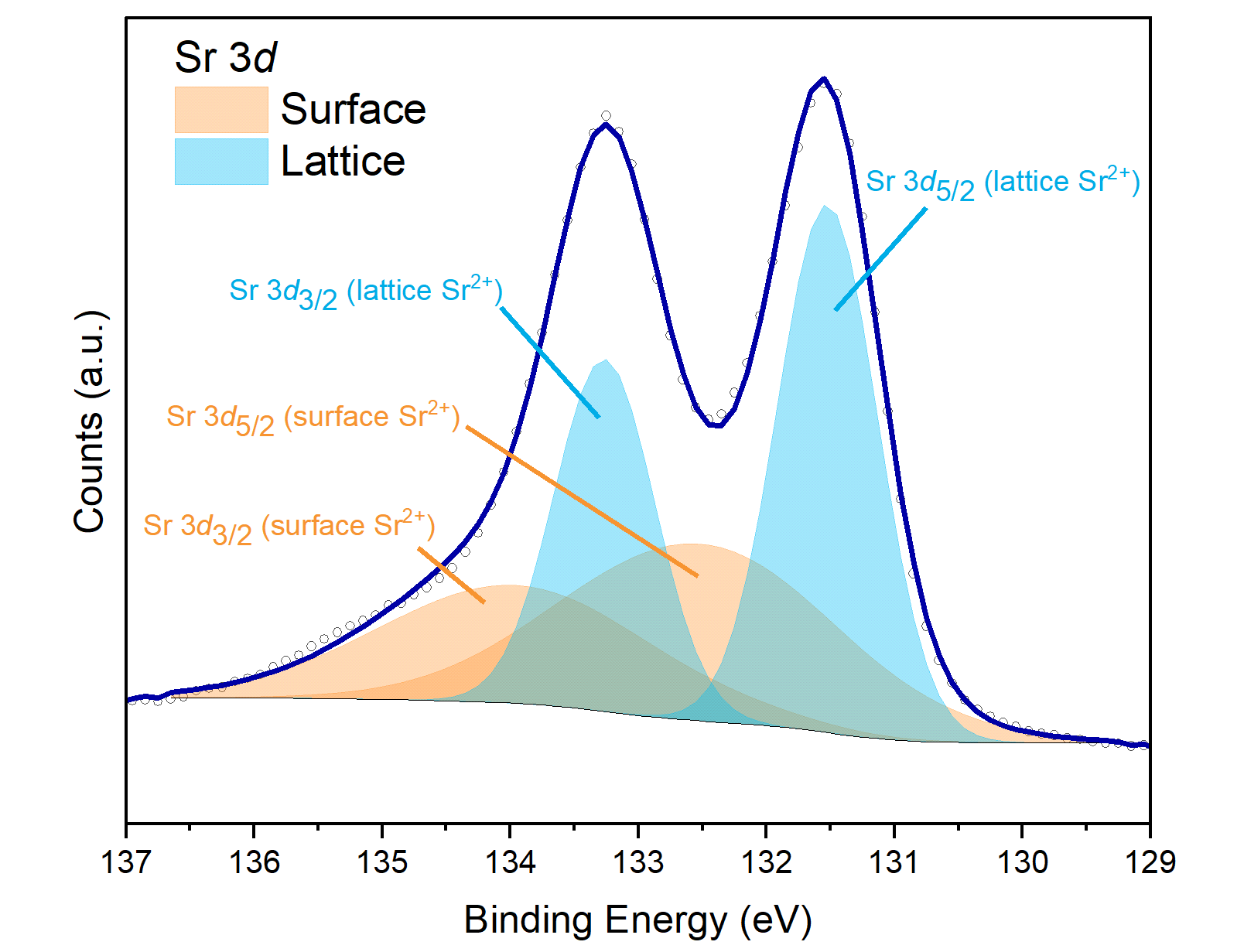


**Figure S6.** An example of fitted Sr 3*d* XPS spectrum of the LSCF sample annealed at 500°C.

**Table S1.** The diffusivity of Sr estimated from the study conducted by Kubicek et al.^2^.

| Temperature (°C) | Bulk diffusivity $\boldsymbol{D}_{\boldsymbol{b}}^{\boldsymbol{Sr}}$  (cm^2^ s^-1^) | Grain boundary diffusivity $\boldsymbol{D}_{\boldsymbol{gb}}^{\boldsymbol{Sr}}$  (cm^2^ s^-1^) |
| --- | --- | --- |
| 350 | 1.2 × 10^-50^ | 8.2 × 10^-55^ |
| 500 | 6.6 × 10^-36^ | 1.2 × 10^-36^ |
| 1250 | 2.9 × 10^-15^ | 3.5 × 10^-11^ |

**Figure S7.** HRTEM images of the LSCF sample annealed at 500°C.

**Section S-6:**





**Figure S8.** SEM image of an APT tip from the LSCF6428 sample annealed at 500°C and subjected to a sharpening process.

**References:**

1. Xu, X., Liu, Y., Wang, J., Isheim, D., Dravid, V.P., Phatak, C., Haile, S.M., *Nature Materials* 2020, **19**, 8.

2. Kubicek, M., Rupp, G.M., Huber, S., Penn, A., Opitz, A.K., Bernardi, J., Stöger-Pollach, M., Hutter, H., Fleig, J., *Physical Chemistry Chemical Physics* 2014, **16**, 6.
